# A physical basis for quantitative ChIP-sequencing

**DOI:** 10.1074/jbc.RA120.015353

**Published:** 2020-09-29

**Authors:** Bradley M. Dickson, Rochelle L. Tiedemann, Alison A. Chomiak, Evan M. Cornett, Robert M. Vaughan, Scott B. Rothbart

**Affiliations:** 1 Center for Epigenetics, Van Andel Research Institute, Grand Rapids, Michigan, USA; 2 Department of Biochemistry and Molecular Biology, Indiana University School of Medicine, Indianapolis, Indiana, USA

**Keywords:** ChIP-Seq, ChIP normalization, quantitative ChIP, antibody specificity, ChIP-sequencing, epigenetics, biophysics, chromatin immunoprecipitation (ChiP), mathematical modeling, quantitative ChIP-Seq, spike-in

## Abstract

ChIP followed by next-generation sequencing (ChIP-Seq) is a key technique for mapping the distribution of histone posttranslational modifications (PTMs) and chromatin-associated factors across genomes. There is a perceived challenge to define a quantitative scale for ChIP-Seq data, and as such, several approaches making use of exogenous additives, or “spike-ins,” have recently been developed. Herein, we report on the development of a quantitative, physical model defining ChIP-Seq. The quantitative scale on which ChIP-Seq results should be compared emerges from the model. To test the model and demonstrate the quantitative scale, we examine the impacts of an EZH2 inhibitor through the lens of ChIP-Seq. We report a significant increase in immunoprecipitation of presumed off-target histone PTMs after inhibitor treatment, a trend predicted by the model but contrary to spike-in–based indications. Our work also identifies a sensitivity issue in spike-in normalization that has not been considered in the literature, placing limitations on its utility and trustworthiness. We call our new approach the sans-spike-in method for quantitative ChIP-sequencing (siQ-ChIP). A number of changes in community practice of ChIP-Seq, data reporting, and analysis are motivated by this work.

ChIP followed by sequencing (ChIP-Seq) was introduced in 2007 (1) as a way to observe the distribution of histone posttranslational modifications (PTMs) and transcription factors (TFs) on the genome. In ChIP-Seq, bulk chromatin is harvested from cells, and an antibody targeting either a TF or a particular PTM is used to collect the subset of chromatin that is bound or cross-linked (2) to the antibody target. The DNA associated with that target-rich chromatin is sequenced, aligned to the host genome, and collected into a histogram. Thus, the distribution of target is measured as a function of genomic location.

Difficulties in reproducibility were forecast at the introduction of the method (1), and the “immunoprecipitation blues” continue to this day (3). Recently, the field has sought to make the method quantitative so that height of the histogram has some physical sense that allows comparison between different experiments and different chromatin samples. A host of methodological alterations have been suggested to establish relative scales (4–9), but all of these require increased complexity in protocol and therefore only increase possible sources for variability, and none call for or facilitate a more robust understanding, practice, or reporting of the method.

What has remained undone until now is the development of a complete, predictive physical model of ChIP-Seq. We show here that by appealing to the physics exploited by ChIP-Seq, a natural framework for quantification, reproducibility, and consistency can be obtained. The standard protocol (10) does not need to be altered to establish a quantitative scale. Moreover, it becomes apparent that ChIP-Seq data reporting is insufficient for understanding variability and reproducibility. Our analysis shows that a number of common measurements made in every ChIP-Seq experiment should be reported, both because they facilitate understanding reproducibility of experiments and because they are required to determine the inherent scale for quantification.

The idea for our approach was to leverage the binding reaction in the immunoprecipitation step of ChIP-Seq to define a physical scale for the sequencing results, allowing comparison of properly designed experiments, and to provide a predictive model for ChIP-Seq outcomes. The quantitative scale for ChIP-Seq arises directly through the existence of the binding isotherm of the IP products. We show example isotherms in Fig. S9. Standard ChIP-Seq involves evaluating only a single point on the isotherm in total neglect of the isotherm generally. Knowledge of the isotherm allows quantitative comparison between ChIP-Seq results for fixed chromatin and different antibody load or fixed antibody load and different chromatin composition (with fixed total chromatin concentration). We focus on the latter in this report.

In the siQ-ChIP context, two or more ChIP-Seq results can be quantified and compared by relationship to the isotherm if the axioms of siQ-ChIP are satisfied by the experimental design: 1) ChIP-Seq IPs must be carried out in equal volumes with 2) equal total chromatin concentration and 3) equal antibody load. If the samples being compared present different epitope distributions, then the product of IP reactions (conforming to the above axioms) can be compared on a quantitative scale without modification of the ChIP-Seq protocol. Below, we define the model, make numerical predictions, and report on application to ChIP-Seq experiments.

## Results and discussion

### A model for generation of sequencing reads

**Figure 1.**
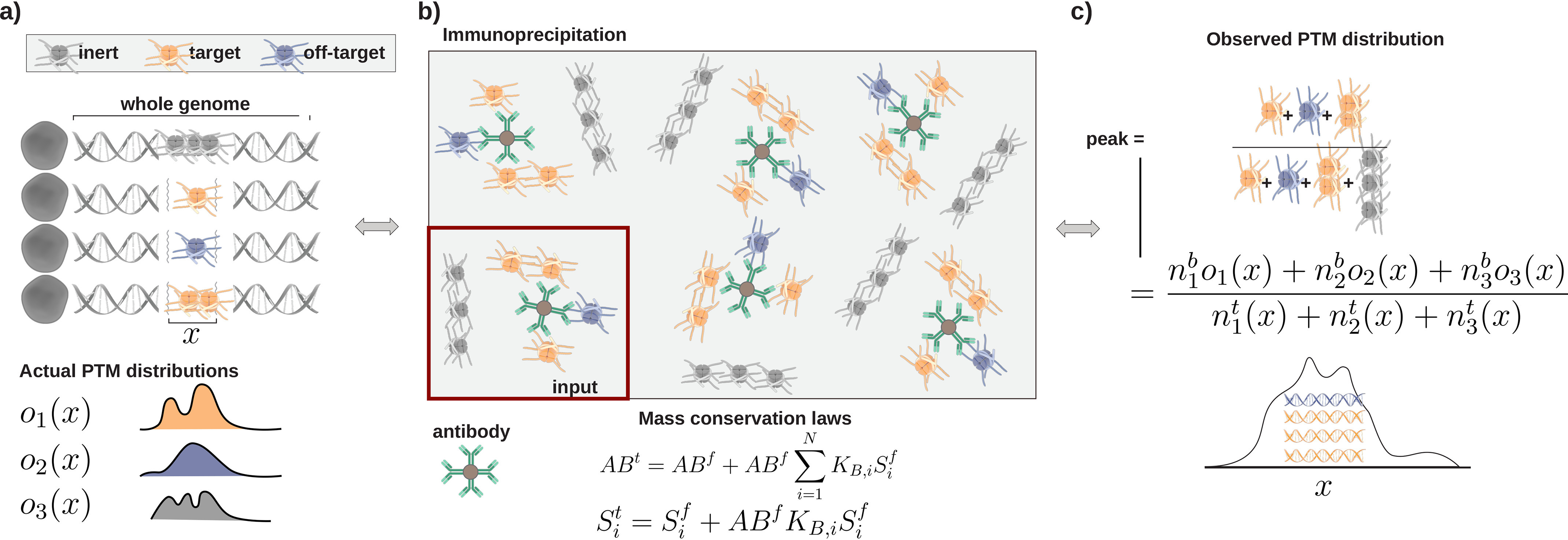
**Schematic of ChIP-Seq.** All *variables* are defined under “Results and discussion.” *a*, cellular chromatin and illustrative species distributions ($o_{i}(x)$) at genomic interval *x*. *b*, immunoprecipitation and input and the mass conservation laws satisfied in the binding reaction. The total mass of antibody and epitope species is conserved in the binding reaction. *AB* and *S*, antibody and epitope concentrations, with superscripts *f* and *t* indicating *free* and *total*. *K_B_* is a binding constant. *c*, illustration of sequencing peak at interval *x*, where *n* is the number of fragments and superscript *b* or *t* indicates *bound* or *total*.

First, note that all sequencing results are the aggregate of sampling the genomes of many cells, as illustrated in Fig. 1*a*. Cellular heterogeneity implies that in a particular genomic interval, *x* different cells can present different target densities or may present an *off-target* that binds to the antibody or an *inert* off-target that does not bind. Thus, when the cellular chromatin is fragmented and captured noncovalently by antibody binding (Fig. 1*b*), both target and off-target fragments spanning *x* are captured, and both types of fragments contribute to the visualized distribution (Fig. 1*c*). Fig. 1*b* describes the antibody capture step, immunoprecipitation (IP), as a competitive binding reaction and is subject to the typical mass conservation laws. These are the conservation laws commonly used to determine binding constants by measuring binding isotherms and fitting. Our interpretation of binding constants is consistent with the treatment of polyvalent systems introduced by Mammen *et al.* (11), as the interaction between chromatin and antibody-bead is of unknown complexity. Application of these laws requires equilibrium, and we report validation of equilibrium for IP in Fig. S8.

With the above discussion in mind, we next define everything in the binding model. The total count of fragments with epitope *i* in interval *x* rendered from C cells is $n_{i}^{t}(x)=\sum_{j=1}^{C}N_{i}^{j}(x)$. The number of fragments of epitope *i* in the interval *x* from cell *j* is $N_{i}^{j}(x)$, although we never need to determine this parameter for each cell. The total number of fragments with epitope *i* in the “multi-cell genome” is $n_{i}^{t}=\sum_{x}n_{i}^{t}(x)$. The fraction of the fragments of epitope *i* at *x* is $o_{i}(x)=\frac{n_{i}^{t}(x)}{n_{i}^{t}}$. The “input” sequencing histogram is then given by the proportionality, $input(x)\propto \sum_{i}n_{i}^{t}(x)$. The “input” is a small-volume aliquot of the chromatin, removed just before the chromatin is reacted with antibody, and is assumed to be a representative sample of all particles present in the intervals *x*. The IP histogram, which is rendered from the antibody-captured subset of chromatin, is $IP(x)\propto \sum_{i}n_{i}^{b}o_{i}(x)$, where $n_{i}^{b}$ is the total number of epitope *i* fragments bound to antibody particles. We express the number of fragments captured on *x* as the expectation against the distribution $o_{i}(x)$, given the assumption that antibody is unbiased with respect to *x*. It is always assumed that the antibody is not biased by genomic location, meaning the antibody has no more preference for target fragments from chromosome 1 than it has for target fragments from chromosome 11. As an example, if the antibody bound to a total of 100 epitope *i*–bearing fragments ($n_{i}^{b}=100$) and only 2% of all *i*-type fragments are at *x* (*o_i_*(*x*) = 0.02), then on average 2 of the 100 bound *i*-type nucleosomes will fall on *x*, $2=n_{i}^{b}\times o_{i}(x)$. Note that nothing has been said about all fragments being mononucleosomal. In the supporting information, we show that the efficiency is computed with fragment length being treated explicitly, allowing for different nucleosome densities to be correctly evaluated.

Intuitively, the chromatin field expresses target “enrichment” as the ratio of sequenced IP fragments (or reads) to sequenced input fragments (or reads) in the interval *x*. Given the formal definitions above, we have the following.

(Eq. 1) $$\frac{\sum_{i}n_{i}^{b}o_{i}(x)}{\sum_{i}n_{i}^{t}(x)}=\text{α}\frac{IP(x)}{input(x)}=\text{α}\frac{\text{IP reads at}\;x}{\text{input reads at }x}$$

The main results of this work are 1) determination of the proportionality constant α and 2) development of a heuristic model for $n_{i}^{b}$ so that we can predict and understand ChIP-Seq outcomes.

Equation 1 states that we expect $IP(x)\propto \sum_{i}n_{i}^{b}o_{i}(x)$, the mapped fragments at *x* are proportional to all of the fragments bound in the IP that map to *x*. There is a similar proportionality expressed for the input. The index *i* runs over every possible interaction captured by the IP. The lowest affinities are expected to be very noisy and strongly perturbed by washes, where high affinities are expected to be easily maintained. In practice, we collect *IP*(*x*) and therefore never specify exactly what all of the values or interpretations of *i* are. In fact, one of the main challenges in ChIP-Seq is gaining confidence that peaks in the interval *x* are actually target peaks. This reflects our lack of practical knowledge for what the index *i* might include. The proportionality constant α is worked out below.

Another technical note about Equation 1 in practice before moving on. We make explicit use of paired-end sequencing in siQ-ChIP. One can use all of the tools of siQ-ChIP for single-end workflows by giving all fragments the same length. However, paired-end has the following advantage that is explicitly utilized in siQ-ChIP quantification. To make the most of having measured the length of a mapped fragment, the length being *L*, we interpret *x* as the genomic interval on which a mapped fragment starts. Keeping track of all of the mapped lengths allows us to write the siQ-ChIP efficiency as follows.

(Eq. 2) $$\hat{e}(x,L)=\alpha \frac{IP(x,L)}{input(x,L)}$$

Both the input and IP lengths are explicit in the efficiency. This provides a significant improvement in information content, as one can see how short and long fragments might be differentially captured. Both a visualization of this two-dimensional efficiency and the details of projecting this to one dimension for visualization in a genome browser are given in Fig. S4.

In the end, the user controls the size of the interval *x* on which the efficiency is computed. This interval has a nonnegligible impact if it is chosen too small. Making the interval too small can result in regions where either IP or input contain mapped fragments but not both IP and input. These regions are evaluated to zero in Equation 2. The interval size should be increased iteratively until it is clear that the results no longer depend on the width of interval. This is shown in Fig. S5.

**Figure 2.**
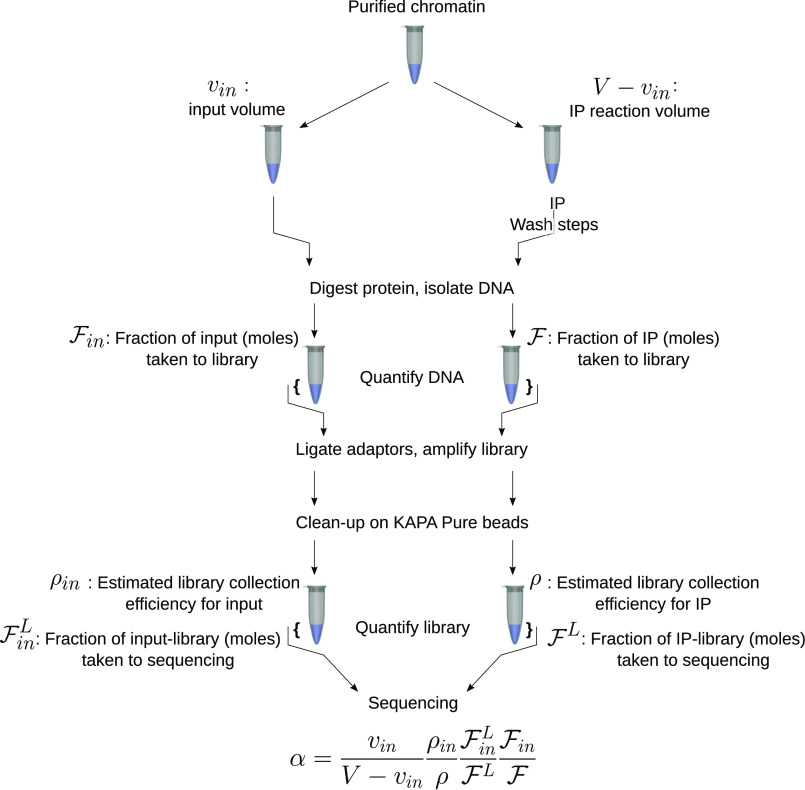
**Schematic deduction of α.** This schematic organizes each factor of α with its origin in the ChIP-Seq protocol. α is the proportionality constant that maintains connection between the material in the IP product and the sequencing reads/fragments.

The proportionality constant α has not been reported in the literature but is straightforward to deduce. Each of the symbols introduced here can be paired with a step of the ChIP-Seq protocol as illustrated in Fig. 2. To deduce α, consider that one of the sequencing experiments reported herein produced a total of $\hat{R}=37,298,373$ mapped IP reads. These reads were generated by sequencing 20 fmol of library, where the total library mass was 856 fmol. Setting $F^{L}=20/856=0.02336$ for the fraction of the library that was sequenced, the total number of reads that the full library would generate upon sequencing is $\hat{R}/F^{L}$, or 1.5 billion for this experiment. The library was amplified with *c* = 11 cycles of PCR, so this estimate of the total reads must be reduced by the appropriate number of amplifications, lowering the estimate of total reads to its preamplification value, $\hat{R}/F^{L}/2^{c}$.^3^ The library was captured on KAPA Pure beads, producing an additional material loss, ρ. ρ is the ratio of captured library concentration to the expected library concentration.^4^ This coefficient compensates for losses due to bead capture and washing and, to some extent, for global deviations from the perfect 2*^c^* amplification. The estimated number of possible reads becomes $\hat{R}/F^{L}/2^{c}/\rho$. The observed read count ($\hat{R}$) has been scaled up by each known source of material loss and down-sampling.

The IP produced 24.2 ng of material, but only 10 ng were used to produce the library, so, where F=0.413 is the fraction of IP material carried into the library, the total fragments generated by sequencing all of the DNA collected by IP are as follows.

(Eq. 3) $$R=\frac{\hat{R}}{F^{L}\;2^{c}\;\rho \;F}$$

Thus, 226 million reads could be extracted from the IP material if all of the material were sequenced. The total sequenced fragments have been scaled to match the total collected fragments, $\sum_{x}\sum_{i}n_{i}^{b}o_{i}(x)$. An analogous scaling also applies to the input sample. The constant α is defined as the ratio of these factors,

(Eq. 4) $$\text{α}=\frac{\rho_{in}}{\rho }\frac{F_{in}^{L}}{F^{L}}\frac{F_{in}}{F}$$

where the subscript *in* refers to the analogous measurements taken on the input sample.

Having established Equations 1, 2, and 4, we have established the requirements for a quantitative ChIP-Seq. (We are assuming that every sequencer is subject to a central limit theorem, which seems implicit in the way sequencing results are currently used. Sequencing results are expected to be reproducible and subject to standard practices for determining means and variances.) One can go one step further now by establishing a predictive heuristic model for the bound particles (or fragments), $n_{i}^{b}$. Any fragments captured by IP are captured because their free energy of binding to the IP beads is sufficiently negative. We take “sufficient” to mean that the interaction survives washes and generates DNA for library preparation. Without specifying any details about what the microscopic state of any of these interactions is, we can associate with each a macroscopic binding constant, *K_B_*_,_*_i_*. This association allows us to specify the following predictive model for understanding ChIP-Seq outcomes.

For any species of epitope *i*, we can write the total concentration in the IP volume as $S_{i}^{t}$. This corresponds to the above definitions as $n_{i}^{t}=(V-v_{in})\;N_{A}\;S_{i}^{t}$, where $V-v_{in}$ is the IP volume, and *N_A_* is Avogadro's number. Thus, chromatin was suspended in a total volume *V*, and then an input aliquot of volume *v_in_* was removed prior to reaction with antibody. The concentration of any bound species can be stated as the difference between total and unbound concentrations, giving us $S_{i}^{b}=S_{i}^{t}-S_{i}^{f}$. Relating back to the definitions above, $n_{i}^{b}=(V-v_{in})\;N_{A}\;S_{i}^{b}$.

Using these definitions and α, Equation 1 can now be recast in terms of epitope concentrations in the IP.

(Eq. 5) $$\begin{array}{ll} \hat{e}(x) & =\alpha \frac{v_{in}}{\left(V-v_{in}\right)}\frac{\text{IP reads at }x}{\text{input reads at }x}\\ & =\frac{\sum_{i}S_{i}^{b}o_{i}(x)}{\sum_{i}S_{i}^{t}(x)}\\ & =\frac{\sum_{i}S_{i}^{t}(\frac{AB^{f}K_{B,i}}{1+AB^{f}K_{B,i}})o_{i}(x)}{\sum_{i}S_{i}^{t}(x)} \end{array}$$

From here on, we include the volume factor in the definition of α. We noted above that one should expect the proportionality $IP(x)\propto \sum_{i}n_{i}^{b}o_{i}(x)$. In Equation 5, we have written $n_{i}^{b}$ as $(V-v_{in})\;N_{A}\;S_{i}^{b}$, which connects the expected outcome of the ChIP-Seq experiment to the binding reaction in IP.

The last line of Equation 5 rewrites $S_{i}^{b}$ as the formal solution to the mass conservation laws in Fig. 1*b*. Details are given in the supporting information. *AB^f^* is the free antibody concentration, determined as the solution to the conservation laws. The bound concentration of each epitope will follow a sigmoidal shape given by y/(1+y), where $y=AB^{f}K_{B,i}$. $K_{B,i}$ is the binding constant for epitope *i*, in the sense of Ref. 11. We note that the above results apply also to sequencing experiments where spike-ins were used. The “genome” in that case is simply understood as the host genome appended with the spike-in DNA so that certain intervals *x* correspond only to spike-in sequences.

### Numerical predictions

In this section, we present simulated outcomes from Equation 5 to make every aspect of the model concrete. The first step of ChIP-Seq is the IP, so this section first covers solutions to the mass conservation equations of Fig. 1*b* to elucidate the composition of bound fragments in the IP for different reaction conditions.

### Binding isotherms

The empirical data presented below pertain to ChIP-Seq outcomes in the case of *epitope depletion*. In that paradigm, a histone PTM, H3K27me3, is depleted by culturing cells in the presence of an inhibitor of the enzyme (EZH2) responsible for chemical addition of the PTM. We will demonstrate below that applying standard ChIP-Seq to a *depleted* and *control* sample results in counterintuitive changes in sequencing peaks, where the *depleted* sample may present larger peaks than the *control* sample (see Fig. 4*a*). No doubt, such observations underlie the broad claims that ChIP-Seq is not quantitative on its own. A quantitative ChIP-Seq would eliminate physically inconsistent outcomes and allow direct comparison of capture efficiency on genomic intervals. We note that an interactive form of the following numerical demonstration is available at the author's web page (www.proteinknowledge.com/siqD3).

**Figure 3.**
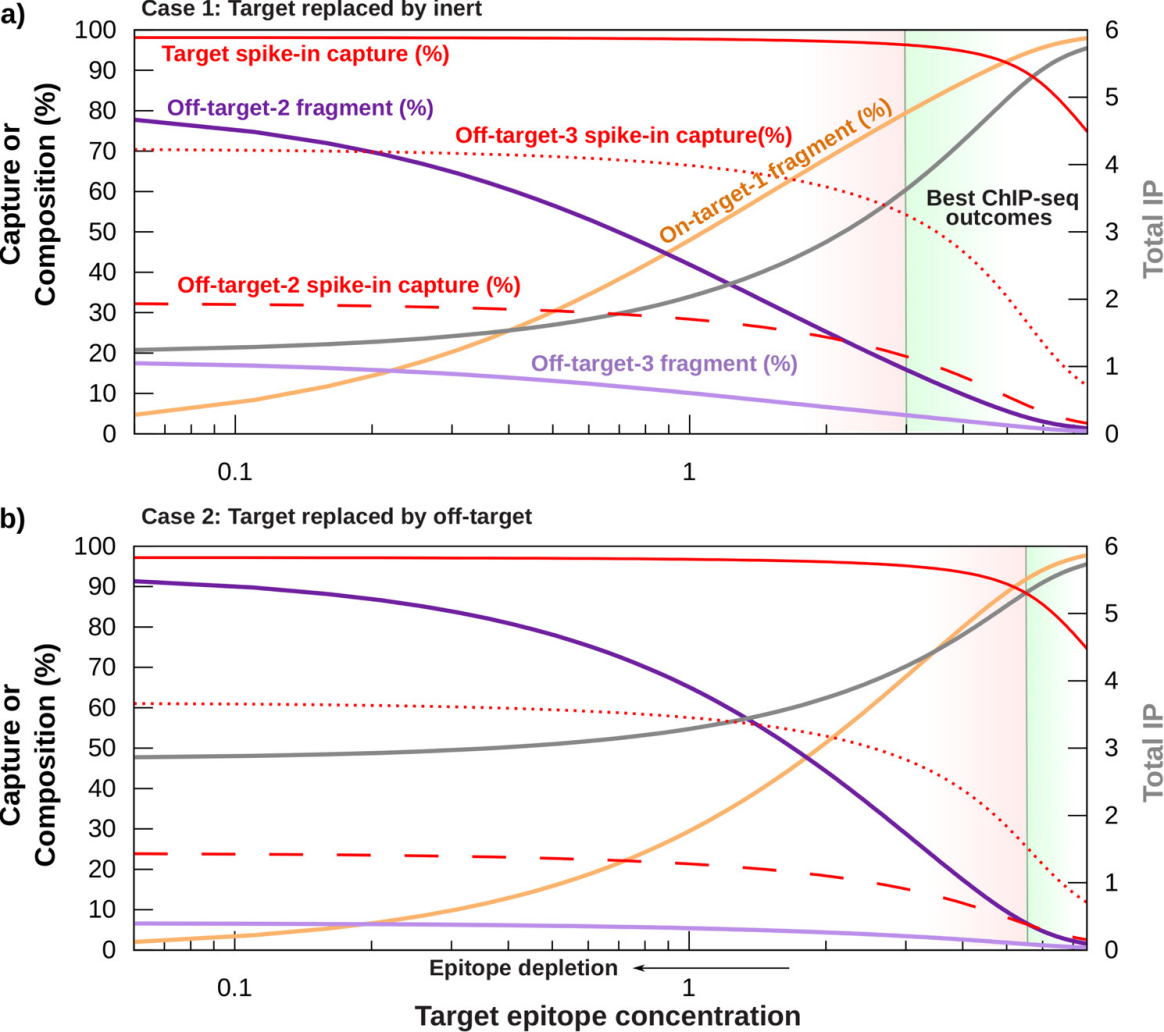
***In silico* predictions for epitope depletion: Sequencing composition in a simulated, four-component system of target (**$S_{1}^{t}$**), off-target (**$S_{2}^{t},\;S_{3}^{t}$**), and inert (**$S_{4}^{t}$**) fragments.** $\%S_{i}^{b}$, percentage of total reads contributed by $S_{i}^{b}$. *a*, **case 1**: target epitope is replaced with inert nucleosomes to model equal chromatin loading in all IP. *b*, **case 2**: target is replaced with off-target, causing $S_{2}^{t}$ to increase while maintaining equal total chromatin loading. At each point in **case 2**, $S_{2}^{t}=11-S_{1}^{t}$ is satisfied. Epitope species 1 is target, species 2 and 3 are off-target, and species 4 is inert. Antibody concentration was 6 μm, and binding constants were $K_{B,1}=11,\;K_{B,2}=0.1,\;K_{B,3}=0.5$ μm^−1^, and $S_{3}^{t}=0.3$ μm. $S_{1}^{t}$, target, was the independent variable. Total concentration bound is shown in *gray* (*right axis*). The inert pool does not interact with antibody and is not shown. These figure panels are broken into *red* and *green zones*. The *vertical black line* separating the zones indicates the break point for potential contamination increase, $S_{1}^{t}=S_{2}^{t}$. This is the point at which the target PTM is no longer the most abundant PTM in the reaction. IP conditions in the *red zones* run greater risk for off-target contamination than conditions in the *green zones*. *Red lines* (*solid*, *dotted*, and *dashed*) show capture efficiency for semisynthetic spike-ins (6, 14).

To understand possible outcome scenarios for a *depletion* experiment, we solved the mass conservation laws in Fig. 1*b* for a four-species system in two physically possible cases. Generally, it is unknown how the chromatin landscape will respond to the use of an inhibitor, so we solved the model for the following scenarios. **Case 1** (Fig. 3*a*) models a system in which epitope is replaced with *inert* fragments. These fragments do not interact with antibody. **Case 2** (Fig. 3*b*) models a system in which epitope is replaced with off-target fragments, causing a net increase in concentration for that off-target PTM. Both cases always present equal total chromatin and antibody concentrations in the IP reaction. Equal chromatin and antibody loading of the IP are the only constraints of siQ-ChIP. To the greatest extent possible, variation in ChIP-Seq outcomes is isolated to perturbation-induced changes in chromatin epitope distribution when this constraint is met.

We do not know binding constants for antibodies, so the simulation assumes that the antibody is 100-fold selective for target $S_{1}^{t}$ over the off-target $S_{2}^{t}$ and 22-fold selective over the remaining off-target $S_{3}^{t}$. Here “selective” is taken as ratios of binding constants. Full details are given in the legend to Fig. 3. Even without exact knowledge of these parameters, this heuristic model allows us to gain intuition for ChIP-Seq outcomes and to make testable predictions for comparison with experiments. Fig. 3 summarizes IP reactions across a range of conditions. Any single experimental outcome would correspond to a single vertical slice of the graphs.

Simulation results in Fig. 3 are plotted as a function of target concentration ($S_{1}^{t}$) and display how the composition of sequencing reads is predicted to change in response to epitope depletion. Epitope depletion is read from Fig. 3 by moving *leftward* along the *x* axis. Importantly, the left-side *y* axis is a percentage, allowing us to plot both the percent composition of bound fragments and the percent efficiency of capture for each species. These are two distinct quantities, and the ChIP-Seq field has so far only considered the capture efficiency because it is easily reflected by spike-in capture efficiency. The key distinction to be aware of is that capture efficiency reflects the fraction (or percent) of a given species that is captured. The fractional composition (or percent composition) reflects how much of the sequenced fragments arise from each species captured in IP. The composition cannot be determined from capture efficiency of spike-ins, but the field has overlooked this reality so far. In Fig. 3 we have plotted spike-in capture efficiency as *red lines* (*solid* or *dashed*), and we have plotted the fractional composition in *solid lines* (*shades* of *purple* for off-target and *gold* for on-target).

There are a few key observations to be made from the results in Fig. 3, which are true of both simulated cases. First, *target depletion results in an increase in the number of off-target reads.* This is seen by looking at the fractional composition of the IP products, shown in *shades* of *purple* (off-target) and *gold* (target). As target epitope is depleted (moving from *right* to *left* in Fig. 3), the fractional composition of target decreases as expected. However, the fractional composition of off-targets increases. So whereas epitope depletion may result in decreasing IP mass, the fraction of that captured mass that belongs to off-target epitope is increasing.

Second, and in stark contrast to the fractional composition, *all species capture efficiencies increase when target concentration is decreased.* The simulated spike-in efficiencies are plotted with *red lines* in Fig. 3. Both target and off-target capture efficiencies increase when the amount of target presented by chromatin is decreased. This is intuitive. The amount of target presented by chromatin is reduced by *depletion*, leaving more antibody to interact with spike-in. Fig. 3 simulates the spike-in consistent with the ICeChIP method (6, 12, 13), where small amounts of synthetic bar-coded nucleosomes are added to the IP. Because these spike-ins are presented in small amounts, the antibody easily saturates them after target *depletion*, leading to an increase in capture efficiency. Remember, the capture efficiency simply reports the fraction of each labeled species that is captured in the IP. Spike-in capture efficiency and unlabeled chromatin capture efficiency are inversely related, yet the spike-in recipe is to normalize to the capture efficiency for target spike-in.

Third, as a corollary to the second observation, spike-in capture will saturate at conditions different from saturation conditions for the unlabeled chromatin. Fig. 3 shows that a large range of IP conditions produce constant spike-in capture efficiency even though the fractional composition of the IP is changing. This means that whereas more and more of the IP product is due to off-target interactions, the spike-ins do not change. Spike-ins are blind to this contamination. A *Drosophila* spike-in may improve sensitivity here (because epitope signals are not separable) but still runs the same risk, especially when the limits of sensitivity for a given IP are not defined. For example, the percentage of on-target reads, or fragments, varies from 90% down to less than 10% in **case 1** of Fig. 3 (*gold line*), whereas the spike-in efficiency only ranges from 90 to 98% for the same experimental conditions. The spike-in capture efficiency is not sensitive to experimental conditions for most of the conditions shown in Fig. 3. Normalizing to spike-ins in these limits would not produce a quantitative scale; the scale would be invariant to the changing amount of target epitope. This limit of invariance can be achieved if antibody is in excess of target and may thus be encountered for tightly distributed PTMs like H3K4me3 (12) or in epitope depletion experiments involving oncohistones (14).

The above observations make the following general predictions for ChIP-Seq outcomes. First, off-target peaks will grow under *depletion* conditions. The extent of growth depends on antibody affinity and homogeneity/heterogeneity of the off-target in the cell population. This growth is predicted even for “selective” antibodies any time the antibody has a nonzero binding constant to any off-target species. Second, spike-in capture efficiencies will be improved by *depletion* for an epitope presenting a nonzero binding constant (that is sufficiently strong to generate sequenced fragments). Finally, we predict that spike-ins have a sensitivity problem that has been ignored in the literature. Any time spike-ins are used, one should validate that they are used in conditions that ensure a measurable response. This is intuitive and obvious but not routinely shown in cases where spike-ins were used. In what follows, we test these predictions in actual ChIP-Seq experiments in the *depletion* paradigm.

### Off-target signal and “specificity”

Before moving on to empirical results, we note that the simulation results pose a problem for the way the community understands “specificity” of antibodies. Typically, off-target reads would be interpreted as on-target reads any time an antibody is deemed “selective” or “specific.” It is a fact that selectivity, even if defined through evaluation of binding (or dissociation) constants for epitopes (15), is a meaningless concept without knowledge of the distribution of epitopes presented by the unlabeled chromatin. Binding constants alone do not indicate the scope of signal contamination. Fig. 3 shows that even for a 100-fold selective antibody, the distribution of epitope in chromatin results in a majority of off-target sequences after target depletion. Moreover, the weaker binding of the off-target epitope, epitope 2, presents larger amounts of bound fragments. This shows that the relative abundance of the epitopes cannot be ignored when attempting to anticipate ChIP-Seq quality. Knowing the binding constants alone would suggest that epitope 3 is more of an issue, but this is not the case. Because the epitope abundances in chromatin samples are generally unknown, there is no way to accurately speculate *a priori* on levels of off-target contamination.

One major advance of the siQ-ChIP approach is that we can leverage the sequencing data directly toward categorizing peaks as on- or off-target. The siQ-ChIP model predicts that individual peaks can be compared for losses and gains in capture efficiency without any traditional “specificity” measurements. For the epitope depletion experiment, the model predicts that off-target peaks will actually grow in height because excess antibody will be free to react. The extent of the increase is controlled by the amount of excess antibody and the strength of interaction between antibody and epitope. Peak heights follow proportionality with the isotherms in Fig. 3. Thus, our predictions for the empirical data below were that the spike-in reagents would show large improvements in capture efficiency for target, some improvement in capture efficiency of off-targets dependent on binding affinities, and increased peak height for any off-target that is capable of capturing excess antibody that was freed by epitope depletion.

The siQ-ChIP efficiency, Equation 5, has in its denominator the sum of all genomic fragments whether or not they are associated with the antibody target and likewise derives its scale from the fact that the IP is a competitive binding reaction. The siQ-ChIP efficiency behaves like the *purple* and *gold lines* in Fig. 3, showing a decrease where target is lost and an increase where free antibody is recruited by off-target PTMs. Note that the species-specific spike-ins show an increase in capture efficiency for spike-in target when target in chromatin is depleted, yet fewer on-target reads are being generated. The simulation trend of increased capture efficiency for off-target spike-ins was also observed empirically for ICeChIP (6) spike-ins (Fig. S1), where target capture efficiency was 8% in DMSO-treated chromatin and 88% in EPZ6438-treated chromatin. (These percentages are computed using α, as described in the supporting information for these spike-ins.)

### Interactive IP and sequencing simulations

To better develop intuition for the siQ-ChIP model, we have built an interactive web page. The page details the model from the perspective of simulating the ChIP-Seq experiment and allows visitors to change parameters and interact with the results. The interactive model can be found at www.proteinknowledge.com/siqD3. In the interactive model, we also present a detailed look at the main challenge facing spike-ins as determinants of quantitative scale: heterogeneity. Because spike-ins and cellular chromatin are mismatched in their respective homogeneity, synthetic spike-ins provide an upper bound for specific PTM capture on any genomic interval.

### Application to ChIP-Seq data

**Figure 4.**
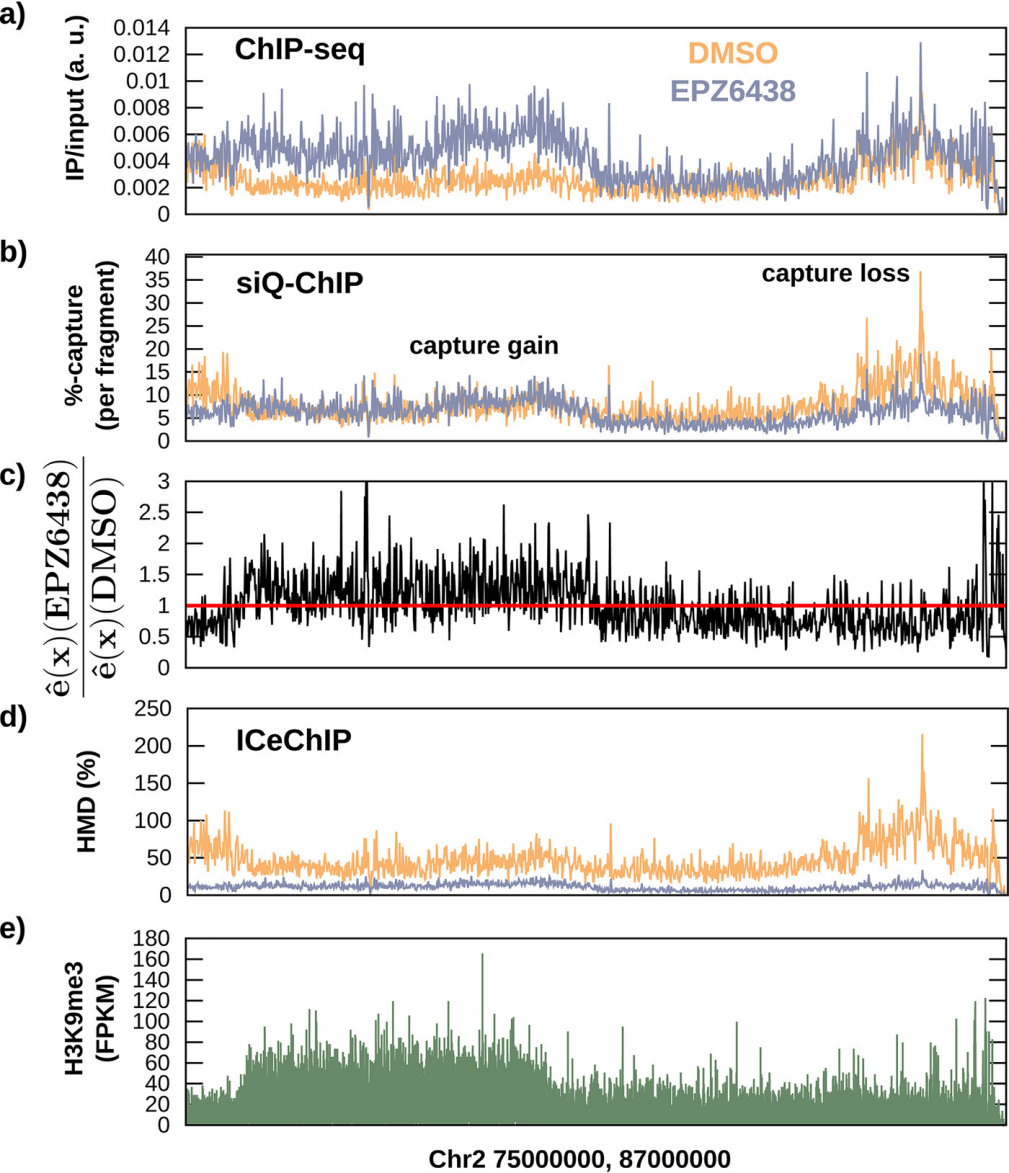
**siQ-ChIP analysis reveals off-target contamination.** *a*, IP/input ratio for unscaled ChIP-Seq data, in units of “efficiency” per base pair. The notion of efficiency in *a* is arbitrary, so arbitrary units are assigned to this *panel*. This *panel* shows that target depletion has produced peaks of unchanged scale and peaks of increased scale, demonstrating the common interpretation that “ChIP-Seq is not quantitative.” See the “Results and discussion” for explanation of this through analysis of α. *b*, the siQ-ChIP capture efficiency per fragment, $\hat{e}(x)\langle L\rangle$. The siQ-ChIP efficiency $\hat{e}$ has units of efficiency per base, and we have multiplied by the average fragment length to produce units of efficiency per fragment. In contrast to unnormalized ChIP-Seq, siQ-ChIP shows peaks of reduced, unchanged, and marginally increased scale. *c*, the siQ-ChIP differential enrichment ratio, EPZ6438/DMSO. This is the ratio of siQ-ChIP efficiencies, which makes it easy to visualize response to target depletion. *d*, HMD from ICeChIP spike-ins shows that the entire signal is reduced throughout the whole genome. The spike-ins “compress,” or shrink, all features of the EPZ6438-treated track. *e*, the H3K9me3 track is shown to suggest that siQ-ChIP is indicating potential off-target capture after EPZ6438 treatment.

To test model predictions described in Fig. 3, we performed native ChIP-Seq for H3K27me3 in HCT116 cells, treated either with DMSO (or “vehicle”) or EPZ6438 (16), an inhibitor of EZH2 (Fig. S7). This is an *epitope depletion* paradigm, wherein the DMSO-treated chromatin represents reaction conditions at the *far right x* axis in Fig. 3, and EPZ6438 treatment shifts those conditions *leftward* along the *x* axis. The antibody target H3K27me3 is globally depleted by exposure to EPZ6438, as evident in Western blotting (Fig. S6), yet by ChIP-Seq there is an apparent increase in peak height (Fig. 4*a*). Fig. 4 reports the results for a 12-megabase stretch of chromosome 2, where Fig. 4*a* shows the ratio of IP/input fragments for standard ChIP-Seq. It can be appreciated that cells treated with EPZ6438 demonstrate peaks on the same scale or even larger than what was seen in cells treated with DMSO. This *panel* illustrates how ChIP-Seq on its own is “not quantitative.” This empirical result for raw ChIP-Seq data is predicted by Equation 5, where the height of IP/input peaks would take proportionality with $\alpha^{-1}$ and direct proportionality with F—as the IP mass decreases, F increases. This is significant in that the emergence or increased height of peaks resultant to cellular perturbations cannot be taken at face value and actually demonstrates a counterintuitive relationship with quantities at the IP.

We also measured α and computed the capture efficiency according to Equation 2 using our open-source software for siQ-ChIP (https://github.com/BradleyDickson/siQ-ChIP). See Equations S17 and S18 for details. The siQ-ChIP results are shown in Fig. 4*b* and demonstrate regions of lost capture, regions of similar capture, and potentially even regions of slight capture efficiency gains.

Fig. 4*b* shows the same IP/input data from Fig. 4*a* scaled by α according to Equation 5. Notice that the EPZ6438-treated chromatin no longer appears to have larger peaks than DMSO. As discussed in the supporting information, the capture efficiency is evaluated in units of efficiency per base pair. Fig. 4*b* uses $\hat{e}(x)\langle L\rangle$, where 〈L〉 is the average base pair length per sequenced fragment, to report efficiency in “per fragment” units. H3K27me3 is largely regarded as broadly distributed so we used Equation S18 to project data into intervals of width 10 kb. At higher resolutions, $\hat{e}(x)\langle L\rangle$ may need to be replaced with the appropriate integral over the distribution of *L*. Fig. 4*c* plots the siQ-differential enrichment as the ratio of $\hat{e}(x)$ for EPZ6438-treated cells to DMSO-treated cells. The enrichment quotient demonstrates regions of impaired and improved capture efficiency.

Fig. 4*d* shows spike-in normalized data generated with the ICeChIP (6) method. The normalization factors in this method are computed simply as the number of IP reads of target spike-in divided by the number of input reads of target spike-in in each treatment, respectively. For DMSO-treated cells, this led to a factor of 1.51, and for EPZ6438-treated cells, it led to a factor of 8.99. Thus, the tracks in Fig. 4*a* are divided by these factors, respectively, and multiplied by 〈L〉 to produce the “histone modification density” on a per fragment basis, as shown in Fig. 4*d*. We note that the values over 100% match the data originally reported for H3K27me3 under histone modification density (HMD) normalization (6). We also note that this normalization demonstrates two different levels of 'background' in the DMSO and EPZ6438 signals, even though these data were produced with identical protocols using the exact same sequencer. The spike-in normalization factors can be compared directly with α^−1^ for the two data sets. For DMSO, the HMD normalization was 1.5, whereas α^−1^ = 9.17. For EPZ6438, the HMD correction was 8.99, whereas α^−1^ = 16.02. As dictated by the definition of α, the ratio of α between the DMSO and EPZ6438 cases is exactly the material difference in the two samples as they arrive at the sequencer, primarily (but not only) due to the mass difference at the IP. The ratio of EPZ/DMSO α values is 1.74, whereas the ratio of DMSO/EPZ IP mass is 2.29. The ratio of HMD normalizations is 5.95. Thus, whereas ratios of α suggest a 2-fold material difference, the ratio of ICeChIP normalizations suggest a 6-fold difference in material. Because α requires tracking all material quantities, we know that a 6-fold difference is not consistent with any measurement made on the samples. (See Table S1.) This speaks to the nonphysical relative scale resulting from spike-in normalization. siQ-ChIP maintains that the reads accumulated on the genome (as shown in Fig. 4) are always connected to the total mass of the IP and input, respectively. This is what establishes a physical scale for the data and is unique from any spike-in approach.

In the previous section, the competitive binding model predicted that both on- and off-target capture efficiencies would increase on epitope *depletion*. Figs. S1–S3 report that spike-in efficiencies improved as predicted after treatment with EPZ6438. Fig. S2 shows that this response in capture efficiency artificially improves the perceived “specificity” of the antibody, meaning that the antibody tests as more specific after target *depletion* when the standard definition of specificity (6, 12) is used. In contradiction with improved “specificity,” the siQ-ChIP model predicts that the quantity of off-target material increases when target is removed, and this is borne out by the raw amounts of captured spike-ins (Fig. S3) as well. Moreover, it is borne out in the genomic sequencing. Fig. 4*c* indicates that there is increased capture of large sections of the genome. Such large regions have been termed mesas by others (17, 18). Using siQ-ChIP, we see that these mesas have increased capture efficiency after epitope *depletion*.

To gain some insight into what these mesas are or might be, we plotted sequencing results from ENCODE (10) for several PTMs alongside our siQ-differential enrichment. Through basic, human-level pattern matching, we identified H3K9me3 antibody tracks as highly correlated with regions of improved capture post-EPZ6438 exposure. This is not too surprising when considering the similarity in histone sequence around Lys-9 and Lys-27 and when considering that we are likely picking up on another broadly distributed (abundant) PTM. Anecdotally, this pattern matching is demonstrated in Fig. 4*e* for this small stretch of chromosome 2. Additionally, we performed an IP using the H3K27me3 antibody followed by Western blotting with an H3K9me3 antibody and found detectable levels of cross-reaction with the IP products. Fig. S6 reports these findings and shows that, by Western blotting, neither H3K27me3 nor H3K9me3 antibody signals are detectable by Western after EPZ6438 treatment.

In summary of Fig. 4, *panel a* demonstrates that “ChIP-Seq is not quantitative.” *Panels b*, *c*, and *e* show that performing ChIP-Seq with an H3K27me3 antibody in epitope-depleted cells (those exposed to EPZ6438) results in a quantitative increase in capture efficiency for genomic regions bearing the H3K9me3 PTM, at least on chromosome 2. *Panels a* and *b* can be interpreted together as evidence that the fraction of captured fragments in the region overlapping H3K9me3 has increased after EPZ6438 exposure, all consistent with our model predictions. The total mass captured by IP has decreased but not vanished (Table S1), also consistent with predictions. The reduction in total mass likely explains the lack of sensitivity by Western blot for EPZ6438-treated chromatin. However, spike-in nucleosomes fail to indicate signal contamination and instead report that the antibody is “specific” in either DMSO- or EPZ6438-treated chromatin (Fig. S2). Despite being reported as “specific” by the accepted metrics, an increase in off-target capture is reported by the spike-ins when target epitope is reduced (Fig. S3), consistent with the model prediction that off-target capture will increase when target epitope is decreased.

**Figure 5.**
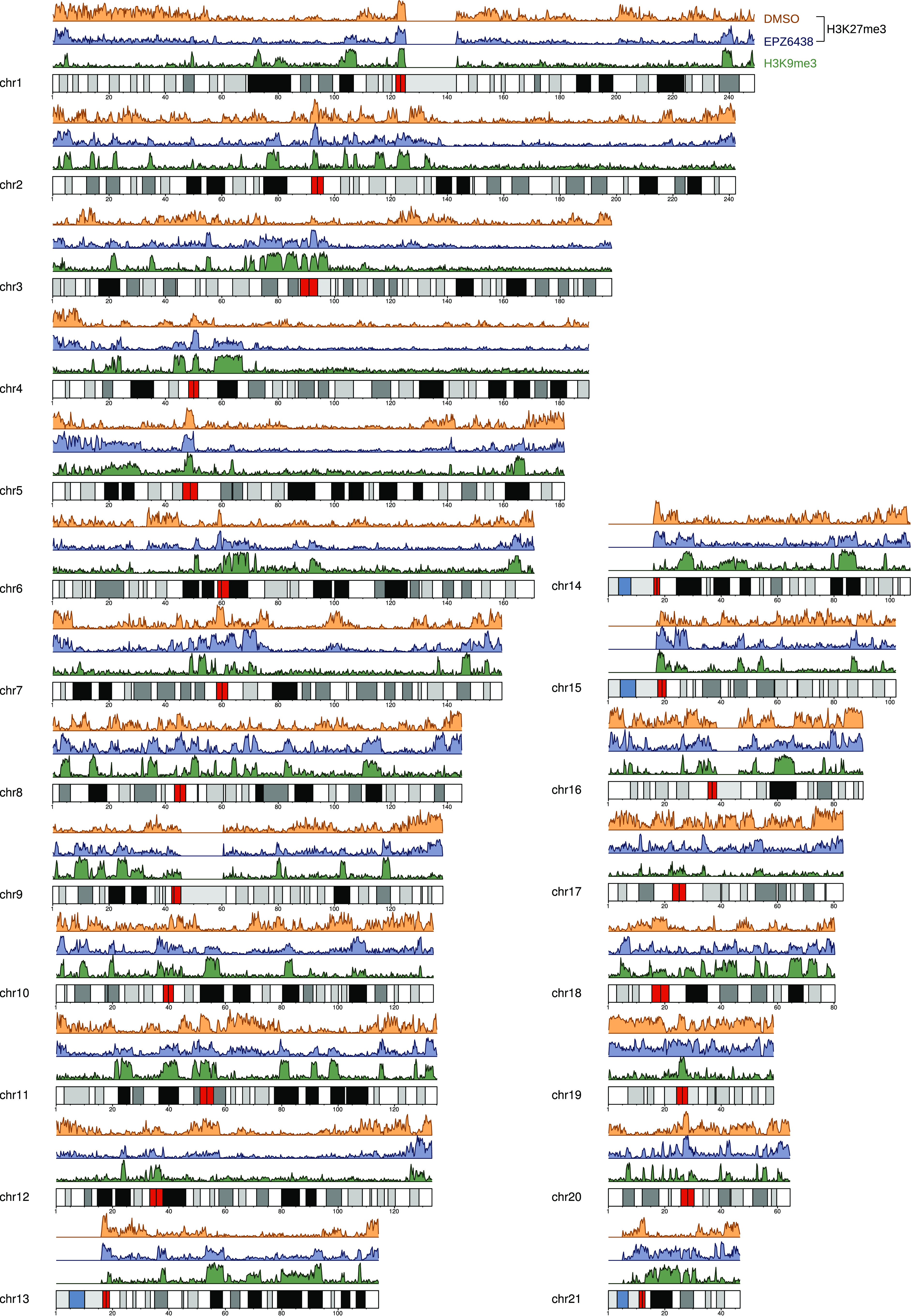
**Autosome peak densities indicating genome-wide off-target contamination.** The peak density (histogram of MACS coverages) on whole chromosomes shows strong correlation with H3K9me3 peak locations. Sequencing data from untreated cells are shown in *orange*, and data from EPZ6438-treated cells are shown in *purple*; both were ChIPs using CST (9773 clone C36B11 lot 14) H3K27me3 antibody. ChIP-Seq from HCT116 cells using Active Motif (39161 lot 1441800) H3K9me3 antibody is shown in *green*. Even though the *purple track* is the result of chipping with H3K27me3 antibody, the sequencing reads are frequently falling on genomic regions of H3K9me3, consistent with predictions from Fig. 3 that off-target will increase contribution to total fragments.

To determine the extent of genome-wide correlation between the sequencing tracks from H3K9me3 antibody in untreated cells and the H3K27me3 antibody–generated sequencing track from EPZ6438-treated chromatin, we called peaks in the raw sequencing data using MACS2 (19) on the DMSO, EPZ6438 (both H3K27me3 antibody), and H3K9me3 antibody (chromatin exposed to neither DMSO nor EPZ6438) data sets. The resulting distributions of called peaks for full human autosomes are shown in Fig. 5. The pattern match between peaks in sequencing from EPZ6438-treated cells and H3K9me3 antibody in untreated cells is easy to appreciate. Thus, we conclude that this experiment demonstrates that either target depletion has resulted in increased off-target binding for the H3K27me3 antibody in EPZ6438-treated cells or there are low-level amounts of H3K27me3 mixed into the putative H3K9me3 mesas. This mixture could be either single histone tails with both Lys-9 and Lys-27 methylation or different tails within the same nucleosome harboring one or the other PTM, or it could be that a subset of cells presents H3K9me3 in the mesas, whereas another subset present H3K27me3 in the mesas. Given the response to target depletion registered by the spike-in nucleosomes (approximately 4-fold for target and less than 1.5-fold for H3K9me3) and the low cross-reaction measured by peptide microarray (20, 21) (Fig. S10), we reason that the small response seen in genomic data suggests that this is indeed off-target cross-reaction. We are also assuming that the EPZ6438 inhibitor is equally effective toward EZH2 inhibition regardless of the genomic region in which EZH2 is found. There is no evidence in the literature to weaken this assumption. We also compared the degree of overlap in H3K9me3 antibody and H3K27me3 antibody coverage for both DMSO and EPZ6438 tracks using the hypergeometric distribution. The overlap is statistically significant in both cases, and the sampling bias increased from 1.33- to 4.19-fold over expected after treatment with EPZ6438. The overlap between H3K9me3 antibody and H3K27me3 antibody tracks increased nearly 4-fold upon epitope depletion, a trend consistent with predictions for off-target response in the heuristic model introduced above.

In summary, it is important to note that the binding model introduced to explain and quantitate ChIP-Seq has predicted the trends of outcomes both for the genomic sequencing and for the spike-ins. Additionally, the evaluation of α has allowed us to measure everything directly within the genomic sequencing, not needing to profile the “specificity” of the antibody. The above discussion used “specificity” profiling to support the likelihood that off-target cross-reaction is being observed only after differential response was measured in the genomic data. In general, we do think such profiling is an affordable way to avoid antibodies of terrible quality—those with roughly equal peptide microarray signals for different PTMs. Nevertheless, we have shown that “specificity” cannot be used to assign confidence to every peak in sequencing outcomes and that rather each peak must be considered individually.

## Conclusion

The above analysis has several consequences. *First, no spike-in is needed to achieve quantification in ChIP-Seq, given adherence to the siQ-ChIP paradigm.* The siQ-ChIP parameter α establishes the natural quantitative scale for ChIP-Seq. It can be argued that spike-ins are only “quantitative” when the spike-in normalizer is equal to α^−1^ for the experiment, a condition that can always be checked. In our experiments above, we showed that not only are the spike-in normalizers different from the physical values of α^−1^, but the ratio of spike-in normalizers produces erroneous interpretations, like a 6-fold difference in apparent mass content. The spike-ins do not produce a quantitative scale in our experiments.

*Second, to improve reproducibility and enable assessment of experimental conditions, all of the values within* α *should be reported for experiments.* This allows the community to compare all of the factors leading up to α and to assess whether repeats are operating in a similar or disparate reaction regime. Given that the IP is a competitive binding reaction, control over these parameters is paramount for reproducibility, yet none of the measurements within α are currently reported. Even reporting only the IP mass and chromatin load in the IP would vastly improve our ability to compare across repeats and afford some ability to mitigate variations in antibody quality. One should match the parameters within α prior to sequencing to view the sequencing results as a “repeat” of the experiment.

*Third, simulations suggest that there are conditions in which spike-ins may not respond to experimental perturbation.* In simulation, this corresponded to conditions of excess antibody, $AB^{t}>S_{1}^{t}$, which might be realized in cases where ChIP-Seq is used to study scarce PTMs or TFs. Our own spike-in target capture improved from 8% to 88% after epitope deletion (Fig. S1), which implies that the spike-in normalization is essentially saturated (Fig. 3). The sensitivity of the spike-in scale is thus dubious.

*Fourth, ChIP-Seq data should always be cross-validated against other available sequencing results for other PTMs (or TFs).* As shown in Fig. 5, ChIP-ing with an antibody against a given target may produce a large number of off-target peaks, depending on the epitope distribution presented by chromatin. Our data show that cross-validation is most important when considering scarce PTMs or epitope depletion like that associated with various inhibition mechanisms, including oncohistones. We will make efforts in the future to automate some cross-validation and statistical assessment of peaks (22).

*Finally, as illustrated in* Fig. 1*c, the histogram measured by ChIP-Seq is not equivalent to the actual distributions of PTMs.* The actual distributions, $o_{i}(x)$ in our notation, underlie ChIP-Seq outcomes, but these signals are convoluted by the superposition of many individual cellular contributions and the imperfect fidelity of the antibody. The extent to which this convolution distorts interpretation is assumed to be small, but this has never been rigorously examined, and single-cell techniques (23) have only recently begun to emerge.

The siQ-ChIP method requires that chromatin and antibody loading be held constant so that changes in chromatin epitope distribution can be isolated and experiments can be understood as motion along the binding isotherm illustrated in Fig. 3. Cases where equal loading produces small IP masses (like hard-to-ChIP TFs or scarce PTMs) are addressed in the supporting information and are fully treatable with siQ-ChIP. The siQ-ChIP scale can be applied to any data retroactively, provided that α can be computed and that chromatin and antibody loading were properly controlled. The values needed for determination of α are not currently reported in the literature, despite the power that these values can afford in understanding variation in repeats (*e.g.* between different laboratories) and interpretation. It is our opinion that even if siQ-ChIP is not used, these values should be reported by practitioners. Table S1 lists each factor in α. Perhaps ironically, all of the measurements required to determine α are made every time ChIP-Seq is performed. However, the measurements are used only for quality control or for meeting sequencing depth requests. Last, we note that results from different sequencers can be compared via siQ-ChIP. siQ-ChIP assumes that the IP and input were sequenced on the same sequencer. Thus, the proportionality constants specific to the sequencer should cancel from the siQ-ChIP capture efficiency. siQ-ChIP should not be used if input and IP are sequenced on different sequencers. The situation of combining IP for sequencing or working with very limited sample amounts is given in Equations S19 and S20.

## Data availability

All data are included in the article and supporting information. All siQ-ChIP codes, scripts, and documentation are published at GitHub (https://github.com/BradleyDickson/siQ-ChIP). Gene Expression Omnibus (GEO) data are accessible under accession number GSE132906.

## Supplementary Material

Supporting Information

## Footnotes

3 If the efficiency ϵ is a known function of sequence x, then amplification can be more accurately accounted for with 1+ϵ(x). Because ϵ is not usually known, we take the typical assumption, 1+ϵ(x)≈2.

4 The average base pair has a molar mass of 660 g/mol. 300-bp fragments are then expected at 198,000 g/mol. If 10 ng of 300-bp-long DNA are amplified by 211 and suspended in 20 μl (our library volume), the expected concentration is 5.17 μm. ρ is the ratio of actual library concentration to the estimated 5.17 μm. In practice, the empirical library averaged fragment length must be used to determine ρ. Thus, average fragment length is an input parameter for the software associated with this work.
