# Supplementary material for: A physical basis for quantitative ChIP-sequencing
Source: J Biol Chem. 2020 Sep 29;295(47):15826–37. doi: 10.1074/jbc.RA120.015353 (PMC7681007; doi:10.1074/jbc.RA120.015353)
Supplement: Supporting Information [file supp_295_47_15826__index.html]

A physical basis for quantitative ChIP-sequencing — A physical basis for quantitative ChIP-sequencing — A physical basis for quantitative ChIP-sequencing — EDITORS' PICK: A physical basis for quantitative ChIP-Seq — Supporting Information 

# A physical basis for quantitative ChIP-sequencing

## Supporting Information

- Supporting Information (to be published online) - Supporting information, two figures were added as noted in the reply letter.
