# Supplementary material for: A physical basis for quantitative ChIP-sequencing
Source: J Biol Chem. 2020 Sep 29;295(47):15826–37. doi: 10.1074/jbc.RA120.015353 (PMC7681007; doi:10.1074/jbc.RA120.015353)
Supplement: Supporting Information [file supp_RA120.015353_162435_1_supp_601795_qh47xl.pdf]

Supplemental Information for: A physical basis for quantitative ChIP-sequencing

Bradley M. Dickson,[1,*] Rochelle L. Tiedemann,[1] Alison A. Chomiak,[1]
Evan M. Cornett,[2] Robert M. Vaughan,[1] and Scott B. Rothbart[1]

[1]*Center for Epigenetics, Van Andel Research Institute, Grand Rapids Michigan 49503, USA.*
[2]*Department of Biochemistry and Molecular Biology,*
Indiana University School of Medicine, Indianapolis, IN, USA.
(Dated: September 23, 2020)

Binding relations for IP and input

For N species that interact with the antibody, the conservation of mass requires that the total antibody concentration (AB^t) equal the sum of free antibody (AB^f) and antibody bound to all the interacting species (S_i)

$$AB^t = AB^f + AB^f \sum_{i=1}^{N} K_{B,i} S_i^f \tag{1}$$

The following constraint also applies

$$S_i^t = S_i^f + AB^f K_{B,i} S_i^f \tag{2}$$

for all N species, where S_i^t and S_i^f are the total and free concentrations of species i, respectively. The free concentrations of each component of the system can be found as the simultaneous solution to these equations if the total concentrations and binding constants are known. In ChIP-seq experiments, nearly none of this information is known, but this model provides relationships that will be useful for ChIP-seq analysis nonetheless.

For each of the $1 \leq i \leq N$ species, the free concentration is

$$S_i^f = \frac{S_i^t}{1 + AB^f K_{B,i}} \tag{3}$$

The concentration of species i that is bound by antibody is

$$\begin{aligned} S_i^b &= S_i^t - S_i^f \\ &= S_i^t \Big(\frac{AB^f K_{B,i}}{1 + AB^f K_{B,i}} \Big) \end{aligned} \tag{4}$$

Equation (4) shows us that the amount of species i that is captured by the antibody depends on the amount of free antibody, which by equation (1), is a function of all the binding constants and concentrations of all of the off-target species. Thus, the amount of bound target epitope depends on the relative amounts of all epitopes that the antibody can bind. Note that equation (4) is the classical logistic function, sometimes called the Langmuir isotherm, associated with binding: When $AB^f = K_d$, exactly half of S_i^t is bound. ($K_d = 1/K_B$ is the dissociation constant.) Thus, we interpret binding constants as macroscopic avidity constants consistent with the formalities of mono- or poly-valent interactions. See for example the development of the enhancement factor β by Mammen, Choi and Whitesides.[1]

The number of moles of species i that were captured can be found by multiplying the concentration S_i^b by the volume of the IP. Let the IP volume be $V - v_{in}$ where v_{in} is a small volume removed prior to introduction of antibody. The volume v_{in} is the input volume. The number of moles captured by IP can be turned into a number of particles using Avogodro's number N_A.

If we assume that every particle of $S_i^b(V - v_{in})N_A$ can generate one read, then the number of reads that S_i^b will generate according to our previous estimates of experimental losses

$$\hat{R}_i = 2^c \, \rho \, \mathcal{F}^L \, \mathcal{F} \, S_i^b \big(V - v_{in}\big) N_A \tag{5}$$

The total reads from IP, $\hat{R}$, is given by adding up the reads $\hat{R}_i$ from each species captured by the antibody. Likewise, the total reads in IP are

$$R = \big(V - v_{in}\big) N_A \sum_i S_i^t \big(\frac{AB^f K_{B,i}}{1 + AB^f K_{B,i}} \big) \tag{6}$$

Through equation (4), the predicted number of reads for each species is rigorously connected to the full spectrum of antibody binding constants, the antibody concentration and the relative concentrations of the various epitopes. The total sequencing reads can now be cast with explicit connection to the parameters governing the antibody binding reaction

$$\hat{R} = 2^c \, \rho \, \mathcal{F}^L \, \mathcal{F} \, \big(V - v_{in}\big) N_A \sum_i S_i^t \big(\frac{AB^f K_{B,i}}{1 + AB^f K_{B,i}} \big) \tag{7}$$

A prediction for the reads generated by i in input can be obtained similarly

$$\hat{R}_i^{in} = 2^c \, \rho_{in} \, \mathcal{F}_{in}^L \, \mathcal{F}_{in} \, S_i^t v_{in} N_A \tag{8}$$

The coefficients appearing here set the natural, quantitative scale in ChIP-seq experiments.

Equations (5) and (8) afford a formal expression for the efficiency of the IP for individual species

$$\frac{S_i^b}{S_i^t} = \frac{\rho_{in}}{\rho} \frac{\mathcal{F}_{in}^L}{\mathcal{F}^L} \frac{\mathcal{F}_{in}}{\mathcal{F}} \frac{v_{in}}{V - v_{in}} \frac{\hat{R}_i}{\hat{R}_i^{in}} \tag{9}$$

This expression for efficiency connects all the reads of species i to the concentration of i that was bound by the antibody, S_i^b, and the total unreacted concentration of i, S_i^t.

Extension to labeled semi-synthetic nucleosomes

Consider projecting equation (9) onto labeled exogenous semi-synthetic nucleosomes. For any nucleosomal species that has a barcoded counterpart, the total species can be broken into the sum of labeled and unlabeled parts $S_i^t = S_i^l + S_i^u$ where l and u denote labeled and unlabeled. The *observable fraction* of S_i^t is $o_i(l) = S_i^l/S_i^t$, which is the labeled (*i.e.,* observable) fraction of the species. Using barcoded spike-ins to report on antibody-chromatin interactions assumes that the interaction with epitopes from labeled and unlabeled (endogenous chromatin) species are the same. Under this assumption, if a total of $\mathcal{N}$ nucleosomes of species i are bound by the antibody in the IP one intuitively expects roughly $o_i(l)$ of those nucleosomes to be labeled. The concentration of bound label can also be found by multiplying equation (4) by $o_i(l)$, $S_i^b(l) = S_i^b o_i(l)$, allowing reads of species i to be projected onto the labeled subset of the species

$$\hat{R}_i(l) = 2^c\,\rho(l)\,\mathcal{F}^L\,\mathcal{F}\,S_i^b\big(V - v_{in}\big)N_A o_i(l) \tag{10}$$

For the labeled input, we have simply

$$\hat{R}_i^{in}(l) = 2^c\,\rho_{in}(l)\,\mathcal{F}_{in}^L\,\mathcal{F}_{in}\,S_i^t(l)\,v_{in}\,N_A \tag{11}$$

because the concentration of labeled spike-in is independent of the unlabeled concentrations. By protocol, the same amount of labeled spike-in is always added to chromatin, so the input cannot report on anything but the labeled concentration which is always the same. The amount of label in the IP, however, is connected to the concentrations of unlabeled chromatin through the competitive binding reaction that is summarized by equations (1) and (2). These estimates of labeled reads can be substituted into equation (9) to find the IP efficiency of the labeled fraction of species i

$$\hat{e}_i(l) = \frac{\rho_{in}(l)}{\rho(l)}\,\frac{\mathcal{F}_{in}^L}{\mathcal{F}^L}\,\frac{\mathcal{F}_{in}}{\mathcal{F}}\,\frac{v_{in}}{V - v_{in}}\,\frac{\hat{R}_i(l)}{\hat{R}_i^{in}(l)} \tag{12}$$

The hat on $\hat{e}_i(l)$ distinguishes the measured efficiency for the label l from the theoretical efficiency e_i for species i. We use $\rho(l)$ to indicate the library losses specifically for the spike-ins. In practice there is no good way to estimate this number and one does not need spike-ins to determine scale so below we willingly take the assumption that $\rho(l) = \rho_{in}(l)$.

Under antibody-saturating conditions, $AB^t < S_i^t$, the reads captured for label l on target species i, $\hat{R}_i(l)$, can be decreased by increasing the unlabeled concentration

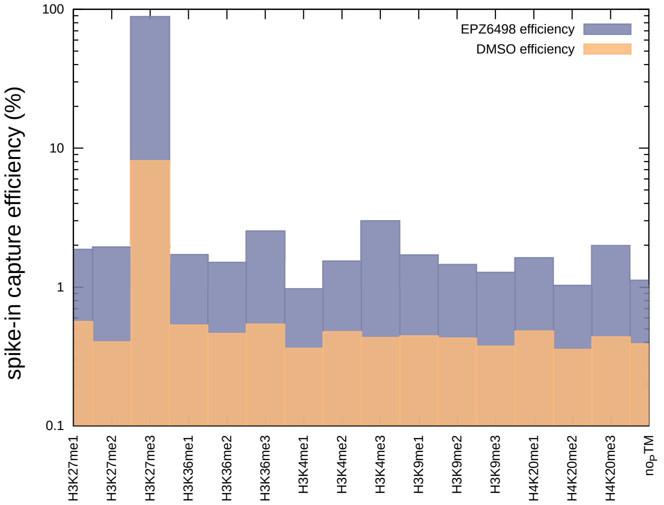

SI-Fig. 1: **Spike-in capture efficiency in ChIP-seq.** Capture efficiencies evaluated by equation (12) for DMSO and EPZ6438 treatments.

S_i^u through the action of $o_i(l)$ in equation (10). In this limit of concentrations the amount of captured particles $S_i^b\,(V - v_{in})\,N_A$ will not change (since all of the antibody is already bound), yet the amount of bound label $S_i^b(l) = S_i^b o_i(l)\,(V - v_{in})\,N_A$ is diminished as S_i^u is increased simply because it is more likely that the antibody encounters unlabeled target. This is intuitive as it reflects the consumption of antibody by increased unlabeled chromatin levels. It is precisely this behavior that disallows the appearance of $o_i(l)$ in the estimate of input reads $\hat{R}_i^{in}(l)$ because the input reads do not depend on S_i^u.

The model, as we have defined $\hat{e}_i(l)$, predicts that the capture efficiency for the on-target H3K27me3 spike-in will approach unity when EPZ6438 inhibitor is used to deplete H3K27me3 levels. The observability of the target label increases with this treatment because the unlabeled concentration of epitope S_i^u decreases, thus S_i^t approaches S_i^l and $S_i^b \to S_i^l$. This prediction was experimentally validated with the spike-in nucleosomes, where we measured a 8% capture efficiency for on-target semi-synthetic nucleosomes spiked in DMSO-treated chromatin versus a 88% capture efficiency when spiked in EPZ6438-treated chromatin. The raw capture efficiency for spike-in nucleosomes is shown in SI=Fig. 1.

To further validate the model with spike-in nucleosomes, additional model-based predictions were tested by experiment. The model affords a predictive framework for several common empirical metrics and how they respond to epitope depletion. Consider the ratio of efficiency for an off-target species j and an on-target species

i

$$\hat{\mathcal{T}}_{j,i} = \frac{\hat{e}_j(l)}{\hat{e}_i(l)} = \frac{\frac{\hat{R}_j(l)}{\hat{R}_j^{in}(l)}}{\frac{\hat{R}_i(l)}{\hat{R}_i^{in}(l)}}$$
$$= \frac{S_j^b o_j(l)}{S_i^b o_i(l)} \frac{S_i^t(l)}{S_j^t(l)} \tag{13}$$
$$= \frac{S_j^b(l)}{S_i^b(l)}$$

Note that the experimental prefactors from equation (12) cancel from numerator and denominator here because $\hat{\mathcal{T}}$ is a ratio of efficiencies. Recall that by protocol $S_i^t(l) = S_j^t(l)$.[2, 3]

The estimator $\hat{\mathcal{T}}_{j,i}$ is used to qualify an antibody as "good," meaning specific, or "bad," meaning non-specific, as follows: For any off-target (j) this estimator is expected to be small for a high quality antibody and comparatively large for a low quality antibody.[2, 3] We used equation (12) in equation (13) to write the specificity estimate ($\hat{\mathcal{T}}_{j,i}$) in two alternate representations that reveal dependence on species concentrations and observability, allowing predictions to be made regarding the behavior of $\hat{\mathcal{T}}_{j,i}$ in the context of epitope depletion. When EPZ6438 is used to deplete H3K27me3, the quantity $S_i^b(l)$ is expected to approach saturation. This is shown numerically in the main text Figure 2. In cells that do not have depleted epitope, $S_i^b(l)$ is predicted to be under saturation. Thus, if the value of $S_j^b(l)$ is roughly independent of the target epitope concentration, as would be expected for weakly interacting off-target species, $\hat{\mathcal{T}}_{j,i}$ is expected to decrease upon epitope depletion by a factor proportional to the increase in the quantity $S_i^b(l)$. That is, an amount proportional to the amount of bound target. Thus, the model predicts a compression of the $S_j^b(l)$ signal reported by $\hat{\mathcal{T}}_{j,i}$ for any off-target j. Reducing S_i^u or increasing S_j^u are predicted to artificially improve observed specificity $\hat{\mathcal{T}}_{j,i}$ without requiring an actual decrease in off-target binding (S_j^b), even though no physical attributes of the antibody have changed. Interestingly, if cellular chromatin presented equal amounts of all PTMs, this signal compression would not be observed, because the factors $o_i(l)$ and $o_j(l)$ would cancel from $\hat{\mathcal{T}}_{j,i}$. Of course, PTMs are distributed very differently, and those distributions change under experimental perturbation.

The other common metric of specificity (in the context of labeled nucleosomes) is

$$\hat{\mathcal{T}}_i = \frac{\frac{\hat{R}_i(l)}{\hat{R}_i^{in}(l)}}{\sum_j \frac{\hat{R}_j(l)}{\hat{R}_j^{in}(l)}} \tag{14}$$

This metric estimates the fraction of on-target signal (or reads) out of the total signal, which includes any off-target reads. This metric is predicted to behave in parallel with $\hat{\mathcal{T}}_{j,i}$ for the same reasons just discussed.

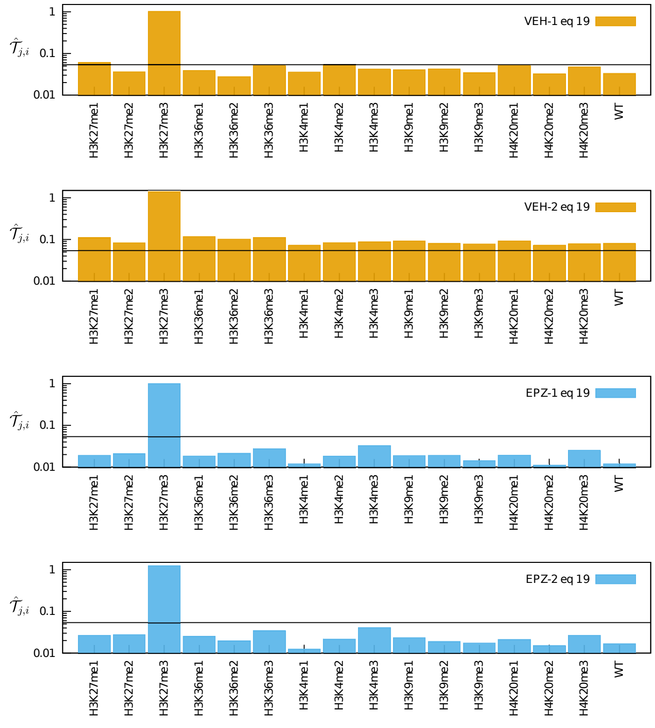

SI-Fig. 2: **Observed selectivity ($\hat{\mathcal{T}}_{j,i}$).** ICe-ChIP nucleosome selectivity profile in ChIP-seq from vehicle (DMSO) or EPZ6438 treated cells, two biological replicates. This is the so-called "% Target" specificity measure. Constant value of 0.05 shown in black as a visual reference. Data is average of ICe-ChIP pairs in one biological replicate. The target epitope, species i, is K27me3. Antibody: CST C36B11 lot 9733S(14)

SI-Fig 2 shows the results of equation (13) for the same antibody in ChIP-seq from either DMSO- or EPZ6438-treated cells. The treatment with EPZ6438 is known to inhibit EZH2 and lead to a global reduction of H3K27me3, constituting widespread epitope depletion. As discussed above, the loss of target epitope reduces S_i^u, where i is the target species, leading to a predicted improvement in observed specificity due to 'signal compression.' Indeed, SI-Fig 2 shows improved specificity for the antibody upon epitope depletion. This shows that the measured specificity is a function of the unlabeled chromatin and does not report on the extent of context independent cross-reactivity of the antibody (*i.e.,* binding constants).

Intuitively, performing ChIP-seq in the presence of excess antibody should increase the likelihood of observing cross reaction between the antibody and off-target species. However, the profiled antibody specificity has improved when antibody is put in excess. The context-dependent variation in measured specificity, and the counterintuitive nature of the estimate, are the source of

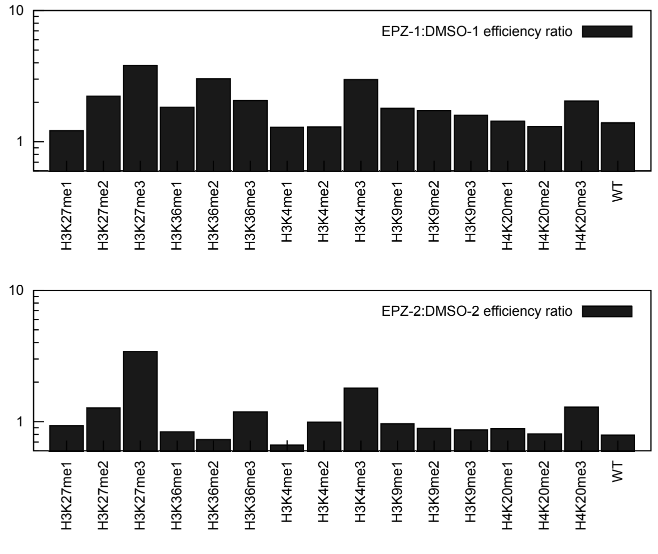

SI-Fig. 3: **EPZ6438-dependent increase in semi-synthetic off-target capture.** Ratio of efficiency, EPZ6438 to DMSO (vehicle), two biological replicates, for each spike-in species.

disparate observations previously reported between peptide array and solution-based assays where the relative amounts of all species are not controlled between assay platforms.[3, 4] This counterintuitive behavior of $\hat{\mathcal{T}}_{j,i}$ was predicted by the model.

SI-Fig 3 shows the ratio of capture efficiency for each species with and without EPZ6438 treatment. This is the capture efficiency of each species compared to itself before and after EZH2 inhibition. In each replicate the capture of off-target species has increased in absolute quantities, as expected, with H3K27me3 depletion. However, this increase is masked in the specificity metrics of $\hat{\mathcal{T}}_{j,i}$ and $\hat{\mathcal{T}}_i$.

Additionally, note that Figure S4B of reference [3] shows that specificity given by equation (14) can be improved from roughly 65% to 80% by adding unlabeled off-target epitope, consistent with the above model-based predictions.

Extension to genomic coordinates

The set of cellular nucleosomes that constitute the particles of species i are scattered throughout the genome. Thus the reads of i are also scattered. Most importantly, reads at a particular genomic coordinate may not arise solely due to binding with a single epitope species. Due to the heterogeneity within a cell population, a mixture of PTMs could arise at a single genomic location, and all nucleosomes at a particular location may not be modified. This section investigates the extension of equation (12) to genomic coordinates in light of heterogeneity.

Let a genomic region be denoted by x, where x specifies the position of an interval of fixed base width. Now x can be thought of in the same way as the label l for exogenous nucleosomes. There is an observable fraction $o_i(x)$ for any species found at x where any of species i that is not at x is understood as unlabeled. The IP efficiency at x is only more complex than it was for synthetic labels because we must sum over all the species present at x to account for all the reads piled up at x. Firstly, the IP reads due to species i is

$$\hat{R}_i(x) = 2^c\,\rho\,\mathcal{F}^L\,\mathcal{F}\,S_i^b\big(V - v_{in}\big)N_A o_i(x) \tag{15}$$

For input we follow all the above arguments to obtain

$$\hat{R}_i^{in}(x) = 2^c\,\rho_{in}\,\mathcal{F}_{in}^L\,\mathcal{F}_{in}\,S_i^t(x)\,v_{in}\,N_A \tag{16}$$

Again, input is not dependent on $o_i(x)$. Second, the total reads at x from IP is given as the sum $\hat{R}(x) = \sum_i \hat{R}_i(x)$. For input, $\hat{R}^{in}(x) = \sum_i \hat{R}_i^{in}(x)$. ChIP-seq experiments only track reads as a function of x and cannot exactly track which species generated the reads.

The reason reads are obtained at x in IP could be through interaction with on- or off-target, or a mixture of both, when population heterogeneity is considered. Thus, the only accessible estimate of IP efficiency is

$$\begin{aligned}\hat{e}(x,L) &= \frac{\rho_{in}}{\rho}\frac{\mathcal{F}_{in}^L}{\mathcal{F}^L}\frac{\mathcal{F}_{in}}{\mathcal{F}}\frac{v_{in}}{V - v_{in}}\frac{\sum_i \hat{R}_i(x,L)}{\sum_i \hat{R}_i^{in}(x,L)}\\ &= \frac{\rho_{in}}{\rho}\frac{\mathcal{F}_{in}^L}{\mathcal{F}^L}\frac{\mathcal{F}_{in}}{\mathcal{F}}\frac{v_{in}}{V - v_{in}}\frac{\hat{R}(x,L)}{\hat{R}^{in}(x,L)}\end{aligned} \tag{17}$$

Two important details must be acknowledged here: First, we are making explicit use of paired-end sequencing which provides the length (L) of each fragment that is mapped to x. Second, in application of this expression, x is the interval within which a fragment starts. We find that $\hat{e}(x,L)$ is only sensitive to the length of the interval x when the interval is taken too small. Basically the data are sparse, limiting the smallest interval width to around 100 base-pair for the sequencing data analyzed in this work. (See SI-Fig. 5) Note that the total reads is now given by $\hat{R} = \sum_x \sum_L \sum_i \hat{R}_i$, which relates everything back to total reads.

Figure 4 shows the capture efficiency as a function of fragment length. The figure clearly demonstrates lost capture efficiency after treatment with EPZ6438, and shows that a number of di-nucleosome sized fragments are lost after treatment.

$\hat{e}(x,L)$ can be transformed for plotting in a genome browser as the cumulative efficiency on an interval y

$$\hat{e}(y) = \frac{1}{\Delta y}\sum_{x,L} 1_{\left[y\cap[x,x+L]\neq\emptyset\right]}\,\hat{e}(x,L) \tag{18}$$

where the indicator function $1_{[\cdot]}$ is unity if the interval y intersects the current fragment and is zero otherwise.

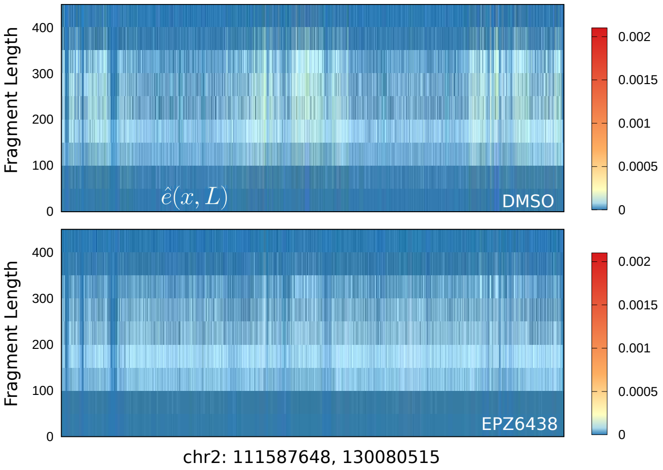

SI-Fig. 4: **Capture efficiency in two dimensions:** The capture efficiency for DMSO and EPZ6438 treated cells is shown. Color is efficiency per base pair.

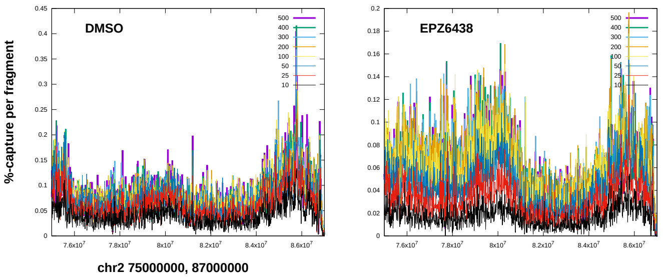

SI-Fig. 5: **Efficiency and parameter dependence** Computing equation (17) for different bin sizes in x and visualized with $\Delta y = 10000$ bp in equation (18).

The length of the interval y in base-pairs is given by Δy so that $\hat{e}(y)$ can be seen as the cumulative efficiency per base-pair. The cumulative efficiency ($\hat{e}(y)$) was shown in Main Text. In Figure 5 we show equation (18) for different bin-widths in equation (17). The results are all self-consistent and parameter-independent for bin-wdiths of at least 100 base pairs. For more narrow bins the data are too sparse and the signal becomes dependent on bin-width.

Before moving away from equation (18), which is the basis of siQ-ChIP, we briefly make note of how our approach interacts with standard peak calling algorithms. There are some deep implications for peak calling, outlined in below, but it is worth noting that siQ-ChIP can be combined with standard peak callers. The widely used MACS peak caller[5], which calls peaks in part by the ratio of IP to input reads can be used here. If MACS is used to call peaks with a threshold $IP/input > \theta$ then the minimal peak height in siQ-ChIP is given by $\frac{\rho_{in}}{\rho}\frac{\mathcal{F}^L_{in}}{\mathcal{F}^L}\frac{\mathcal{F}_{in}}{\mathcal{F}}\frac{v_{in}}{V-v_{in}}\theta$. Any hidden Markov model peak caller would also be suitable. It is suggested to call peaks on the underlying data and then use those locations to examine/process $\hat{e}(x)$. However, as shown in Main Text, it is completely reasonable to plot the ratio of cumulative efficiency (equation (18)) from two experiments that are being compared. This provides immediate access to regions of differential enrichment without using any peak callers. The chromatin and antibody concentrations, as well as reaction volume and buffers, must all be held constant. Changes in sequencing are then isolated to changes in the distribution of epitope in chromatin.

Flexible protocols

For hard to ChIP PTMs it may be impossible to obtain a workable amount of DNA from one IP reaction. It is possible to combine M IP reactions, where each IP is required to have identical reaction conditions, as follows

$$\begin{aligned}\hat{R}_i &= 2^c\,\rho\,\mathcal{F}^L\,\mathcal{F}\sum_{IP}^{M}\left[S^b_{i,IP}(V-v_{in})N_A\right]\\ &= 2^c\,\rho\,\mathcal{F}^L\,\mathcal{F}\,M\left[S^b_i(V-v_{in})N_A\right]\end{aligned} \quad (19)$$

S^b_i is replaced by the average of $S^b_{i,IP}$ over the M IPs. Since $S^b_i = \frac{1}{M}\sum_{IP} S^b_{i,IP}$, these two representations are equivalent. In practice this assumes that each Mth IP is perfectly well sampled, *e.g.,* the distribution of species in the IPs are the same as the distribution of species in the average S^b_i. Formally, the practice of using less than the full IP mass in sequencing (in our case the IP mass was brought down to 10 ng) is equivalent to making this assumption on the distribution of species. It is assumed that the 10 ng fraction is equal in species distribution to the full IP mass.

Recognition of the assumptions invoked upon combining IP allows us some additionaly flexibility in siQ-ChIP. Suppose we have two samples that we need to compare. We have enough chromatin from each sample so that we can setup IPs at equal chromatin and antibody loads. One sample generates an IP mass $\mathcal{I}$ and the other an IP mass $\mathcal{J}$. Further suppose these samples generate less than 10 ng of DNA and are too rare to allow multiple IP. In each case, the full IP mass is taken into sequencing so that $\mathcal{F} = 1$ The above formalities of combining repeat IP can be used here to set $M = \mathcal{I}/\mathcal{J}$, where now M is the number of IP that must be performed to bring the IP mass $\mathcal{J}$ up or down to match the mass $\mathcal{I}$. This value of M can be used in equation (20) below for computing capture efficiency for sample $\mathcal{J}$. Now the two rare samples can be correctly compared even though we cannot obtain the exact same IP mass for each sample. The only assumption required is that repeats of the IP are indeed drawn from the same distribution of outcomes.

Using the above adjustment in the effective efficiency only modifies the siQ-ChIP capture efficiency by the factor $\frac{1}{M}$. This result is intuitive, as it clearly maps the

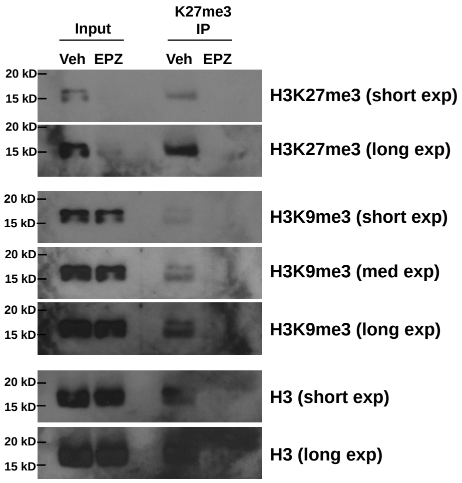

SI-Fig. 6: **EPZ6438 causes global reduction of H3K27me3:** Chromatin from DMSO (Veh) and EPZ6438 (EPZ) treated cells was harvested and IP'd with the H3K27me3 antibody. The IP'd material was also blotted for H3K9me3. Treatment with EPZ6438 can be seen to reduce antibody capture to less than detectable levels.

results on to a per-IP basis. Explicitly, the effective efficiency becomes

$$\hat{e}(x,L) = \frac{1}{M}\frac{\rho_{in}}{\rho}\frac{\mathcal{F}_{in}^{L}}{\mathcal{F}^{L}}\frac{\mathcal{F}_{in}}{\mathcal{F}}\frac{v_{in}}{V - v_{in}}\frac{\hat{R}(x,L)}{\hat{R}^{in}(x,L)} \tag{20}$$

Obviously, normalization to cell number is encoded here.[6]

IMMUNOPRECIPITATION FOLLOWED BY WESTERN BLOT

In 6 we confirm global H3K27me3 loss by western blot and we demonstrate cross-reaction between the IP products of the H3K27me3-antibody and the H3K9me3-antibody. The source of the cross-reaction is unknown, although by peptide microarray there is not significant direct cross-reaction predicted for the respective H3K27me3 and H3K9me3 targets.[7] However, the concentration of these species is different in cellular chromatin and microarray. (See www.histoneantibodies.com for antibody array screen.) The detection limit in this assay prevents us from making observations after treatment with EPZ6438.

EXPERIMENTAL METHOD DETAILS

Native Chromatin Immunoprecipitation

Cell Culture:

HCT116 colorectal carcinoma cells were cultured in McCoy's 5A supplemented with 10% FBS and 1% penicillin-streptomycin and treated for 72 hours with either vehicle (0.02% DMSO and 0.03% PBS) or EZH2 inhibitor (EPZ6438, 1 uM). Cells were trypsinized, collected, flash frozen, and stored at -80°C.

Nuclei purification:

To purify nuclei, thawed cells were washed 3x with PBS, 2X with Buffer N (15 mM Tris buffer pH 7.5, 15 mM NaCl, 60 mM KCl, 5 mM $MgCl_2$, 1 mM $CaCl_2$, 8.5% sucrose, 1 mM DTT, 200 uM PMSF, 50 ug/mL BSA, and protease inhibitor), and lysed with 2X Lysis Buffer (0.6% NP-40 in Buffer N). Nuclei were layered over a 30% sucrose cushion, pelleted at 1300 rcf, and resuspended in Buffer N.

Nucleosome preparation:

Crude chromatin concentration was determined by sonicating 2 uL of nuclei in 18 uL NaCl (2 M) and measuring DNA concentration with a Nanodrop; nuclei equivalent to 50 ug of DNA were aliquoted and spiked with 5 uL of a SNAP-ChIP k-MetStat panel (EpiCypher). 1 U MNase (25 U/uL) was added per 4.3 ug chromatin and incubated at 37°C for 12 minutes with shaking. MNase digestion was stopped with 1/10 volume MNase Stop Buffer (10X, 0.1 M EGTA) followed by 1/8 volume NaCl (5 M) to lyse nuclei and release digested chromatin. After centrifugation, soluble chromatin was added to 33 mg of rehydrated ceramic hydroxyapatite (CHT) resin and rotated for 10 min at 4°C. The CHT Resin:Chromatin mix was added to a centrifugal filter unit and washed 4X with HAP Wash Buffer 1 (5 mM $NaPO_4$ pH 7.2, 600 mM NaCl, 1 mM EDTA, and 200 uM PMSF), 4X with Hap Wash Buffer 2 (5 mM $NaPO_4$ pH 7.2, 100 mM NaCl, 1 mM EDTA, and 200 uM PMSF), and eluted with three successive additions of HAP Elution Buffer (500 mM $NaPO_4$ pH7.2, 100 mM NaCl, 1 mM EDTA, and 200 uM PMSF). DNA concentration was again measured (as above) and adjusted to 20 ug/mL with ChIP Buffer 1 (25 mM Tris pH 7.5, 5 mM $MgCl_2$, 100 mM KCl, 10% glycerol, 0.1% NP-40, 200 uM PMSF and 50 ug/mL BSA).

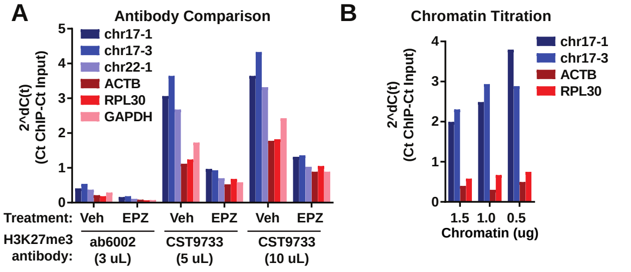

SI-Fig. 7: **ChIP optimization.** A) ChIPs from DMSO and EPZ6438 treated samples (chromatin equivalent to 3 ug DNA) using Abcam (ab6002) and Cell Signaling Technologies (CST #9733) H3K27me3 antibodies were compared. H3K27me3-positive (blue: chr17-1, chr17-2, chr22-1) and negative regions (red:ACTB, RPL30, and GAPDH promoters) were tested by qPCR. B) Different concentrations of chromatin equivalent to 1.5, 1.0, and 0.5 ug DNA were immunoprecipitated with CST #9733 (5 uL). Average of technical duplicates plotted for each.

Antibody:bead preparation:

Antibody:bead conjugates were prepped by adding 5 or 10 uL of Cell Signaling Technology H3K27me3 antibody (CST #9733, clone C36B11, lot 14, 102 ug/mL) or 3 uL of abcam H3K27me3 antibody (ab6002, 1 mg/mL) to 12.5 uL of Protein A Magnetic Dynabeads (Invitrogen) that was prewashed washed with ChIP Buffer 1. 5 uL of CST #9733 was chosen as the optimal antibody after comparison with ab6002 (Figure 7A). Antibody:bead conjugates were incubated on a rotator for 3 hours at 4°C, washed 2X, and resuspended with ChIP Buffer 1.

Chromatin Immunoprecipitation:

Between 0.5 and 3 ug of chromatin was used for optimization (Figure 7B). Based on this, 0.75 ug of the purified digested chromatin (as measured by DNA concentration) was added to the antibody:bead conjugates while a volume equivalent to 10% was saved for input from the purified chromatin. The bead:antibody:chromatin mixture volume was brought to 100 uL with ChIP Buffer 1 and incubated on a rotator for 17 min at 4°C. Using a magnetic rack, the mixture was washed for 10 min on a rotator at 4°C 2X with ChIP Buffer 2 (25 mM Tris pH 7.5, 5 mM $MgCl_2$, 300 mM KCl, 10% glycerol, 0.1% NP-40, 200 uM PMSF and 50 ug/mL BSA), 1X with ChIP Buffer 3 (10 mM Tris pH 7.5, 250 mM LiCl, 1 mM EDTA, 0.5% sodium deoxycholate, 0.5% NP-40, 200 uM PMSF and 50 ug/mL BSA), 1X with ChIP Buffer 1, 1X with TE Buffer (pH 8.0), and resuspended in 50 ul of ChIP Elution Buffer. This was incubated at 55°C for 5 min, and sample was eluted from beads on a magnetic rack. Finally, 2 uL NaCl (5 M), 1 uL EDTA (0.5 M), and 0.5 uL Proteinase K (20 mg/ml stock) were added to ChIP and Input samples, which were incubated overnight at 55°C.

Purification of Immunoprecipitated DNA:

DNA was recovered using KAPA Pure beads at a 1.5X ratio on a magnetic rack. After two washes with 75% EtOH, DNA was eluted in 50 uL dH20 and DNA concentration was measured with a Qubit. Input and ChIP Libraries were prepared from 10 ng DNA using a KAPA Hyper Prep Kit. Libraries were purified once more with KAPA Pure beads at a 1.0X ratio to remove adapter contamination.

Cross-linking Chromatin Immunoprecipitation:

Cell Fixation and Collection: Approximately 10 million HCT116 cells were grown to 80% confluency in a 10 cm plates. Cells were washed in the plate with 5 mls of 1X PBS at room temperature. The 1X PBS wash was removed, and 5 mls of Fixing Buffer (50 mM HEPES-KOH pH 7.6, 100 mM NaCl, 1 mM EDTA pH 8.0, 0.5 mM EGTA pH 8.0) was added. Cells were fixed by adding 313 ul of freshly prepared 16% methanol-free formaldehyde solution (Thermo Scientific Catalog #28906) to a final concentration of 1%, and cells were incubated on a shaker at room temperature for 10 minutes. Formaldehyde was quenched by adding 266 ul of 2.5 M Glycine (final concentration 125 mM), and cells were incubated for an additional 5 minutes on the shaker at room temperature. Cells were collected from the plate by scraping the monolayer and diluting the fixation solution with 10 mls ice-cold 1X PBS. Cells were pelleted at 200 x g for 5 minutes at 4°C. Cells were washed twice more with 5 mls ice-cold 1X PBS and collected by centrifugation at 200 x g for 5 minutes at 4°C. The final PBS wash was carefully aspirated from the cell pellet, and cells were flash frozen in liquid nitrogen and stored at -80°C until use.

Nuclei isolation and sonication: Cells were thawed on ice for 10 minutes prior to cell lysis. Cells were lysed in 1 ml LB1 (50 mM HEPES-KOH pH 7.6, 140 mM NaCl, 1 mM EDTA, 10% Glycerol, 0.5% NP-40, 0.25% Triton X-100, Protease inhibitor cocktail (Roche cOmplete Mini tablets, EDTA-free) and incubated for 10 minutes rotating at 4°C. Intact nuclei were collected by centrifugation at 1,700 x g for 5 minutes at 4°C. Supernatant was removed so as not to disturb the nuclei pellet, and nuclei were resuspended and washed in 1 ml LB2 (10 mM Tris-HCl pH 8.0, 1 mM EDTA, 0.5 mM EGTA, 200 mM NaCl, protease cocktail inhibitor) for 10 minutes rotating at 4°C. Nuclei were collected by centrifugation at 1,700 x g for 5 minutes at 4°C, and the supernatant was removed so as not to disturb the nuclei pellet. Finally,

nuclei pellets were gently rinsed twice without disturbing the pellet with 1 ml LB3 (10 mM Tris-HCl pH 8.0, 1 mM EDTA, 0.5 mM EGTA, 0.01% NP-40, protease cocktail inhibitor) and collected at 1,700 x g for 5 minutes at 4°C. Following the two rinse steps, nuclei were resusented in 1 ml LB3 and transferred to a 1 ml milliTUBE (Covaris) for shearing. Nuclei were lysed and chromatin was sheared to range of 300-600 base-pair fragments using a Covaris E220 evolution Focused ultrasonicator with the following parameters: Peak power (140.0), Duty Factor (5.0), Cycles/Burst (200), Duration (600 seconds), Temperature (4°C).

Immunoprecipitation: Sheared chromatin was quantified by Bradford Assay, and 300 ug of chromatin was brought to a final volume of 500 ul in LB3, and then an additional 500 ul of ChIP Cocktail Mix (40 mM Tris-HCl pH 7.6, 150 mM NaCl, 1 mM EDTA pH 8.0, 1% Triton X-100, 0.5% NP-40, Protease inhibitor cocktail) was added to bring the final volume to 1 ml. Prepared chromatin was then pre-cleared by incubation with 20 ul of pre-washed Dynabeads Protein G magnetic beads (Invitrogen Catalog #:10004D) for 3 hours at 4°C with constant rotation. Prior to incubation with antibody, 10 ul of pre-cleared chromatin was removed and set aside to serve as 1% input. Pre-cleared chromatin was removed by magnetic separation, transferred to a new tube, and immunoprecipitated with 5 ul of H3K9me3 antibody (Active Motif 39161, Lot# 14418003) overnight at 4°C with constant rotation. Protein G magnetic beads (35 ul/IP) were blocked overnight in 1 ml of 1X PBS, 0.5% BSA, and 20 ug of Herring Sperm DNA (Sigma Catalog #D7290) at 4°C with constant rotation. The next morning, blocked beads were washed three times with 1X PBS + 0.5% BSA, and two times with WB1 (50 mM Tris-HCl pH 7.6, 150 mM NaCl, 5 mM EDTA pH 8.0, 0.5% NP-40, 1% Triton X-100). Immuno-chromatin complexes were incubated with blocked beads for 3 hours at 4°C with constant rotation. Unbound chromatin was then removed using magnetic separation, and the beads were washed as follows: 3 times with WB1, 3 times with WB2 (50 mM Tris-HCl pH 7.6, 500 mM NaCl, 5 mM EDTA pH 8.0, 0.5% NP-40, 1% Triton X-100), 2 times with WB1, and 1 time with Low Salt TE (10 mM Tris-HCl

pH 8.0, 1 mM EDTA pH 8.0, 50 mM NaCl). All washes require 5 minute incubations at 4°C with constant rotation followed by magnetic separation and removal of buffer.

Elution and DNA clean-up: To elute DNA from the magnetic beads, 50 ul of Elution Buffer (10 mM Tris-HCl pH 8.0, 10 mM EDTA, 150 mM NaCl, 5 mM DTT, 1% SDS) was added to the beads and incubated at 65°C for 15 minutes. The elution step was repeated, and eluates combined. Eluents and 1% input (with 90 ul of elution buffer added) were incubated overnight at 65°C with constant shaking to reverse cross-link protein:DNA complexes. The next morning, 2 ul of DNase-free RNase A (10 mg/ml stock) was added to eluents and incubated at 37°C for 1 hour. Next, 10 ul of Proteinase K (20 mg/ml stock) was added to eluents and incubated at 37°C for 2 hours. DNA was isolated following standard KAPA Pure Beads (KAPA Biosystems Catalog# KK8000) protocol with a 1.5X ratio of beads to DNA volume as described for the Native Chromatin Immunoprecipitation. Final elution was in 20 ul of Nuclease-free water, and DNA concentration was measure by Qubit dsDNA High Sensitivity Assay kit (ThermoFisher Scientific Catalog#: Q32851). Library preparation was done as described for Native Chromatin Immunoprecipitation.

Sequencing:

Prepared libraries were submitted to the VARI Genomics Core for library QC and quantification using the Agilent 2100 Bioanalyzer and KAPA Library Quantification Kit, respectively. Libraries were sequenced on an Illumina NextSeq 500 with 2x75 paired-end reads.

NGS Data Processing:

Adapters were trimmed from fastq sequences using TrimGalore! version 0.5.0 Trimmed sequences were queried for overall sequencing quality using FastQC version v.0.11.8 Sequences were then aligned to the human hg38 genome build using the following command in bowtie2 version 2.3.4.3 ([8]):

```
  bowtie2 -I 0 -X 700 --end-to-end --sensitive
-x
  /path_to_hg38_index -1
  /path_to_first_paired_read.fastq.gz
  -2 /path_to_second_paired_read.fastq.gz
  -S sample.sam
```

SAM files were further processed with the following command to isolate paired reads with high mapping quality, correct pair orientation, and to calculate fragment length:

```
  awk -v MAQ=20 $5>=MAQ && $2==99
  || $5>=MAQ && $2==163 {print
    $3''\t''$4''\t''$4+$9-1}
  sample.sam | awk '$2<=$3
  {print $1''\t''$2''\t''$3"\t"$3-$2}' |
  sort -k1,1 -k2,2n > outfile.bed
```

Finally, known blacklisted regions were removed from the bed files using the subtract function from bedtools. ([9, 10])

MACS2 peak calling:

To call peaks of modification enrichment for both our H3K9me3 ChIP-seq and H3K27me3 ChIP-seq datasets, we used the following command from macs2 ([5]) (v2.1.2):

```
  macs2 callpeak -t {IP_sample}.bam -c
  {Input_sample}.bam -f BAMPE -n {SAMPLE}
-outdir
/path_to_output_dir/ --broad -B
```

Bedtools intersection:

To determine the coverage of the genome (in bp) that intersected between two datasets we performed the following command in bedtools ([10]) for the different datasets:

```
bedtools intersect -a dataset1.bed -b
dataset2.bed -wo | awk '{ sum +=
  $column_with_overlap_bp_count; } END { print
sum; }' ''$@''
```

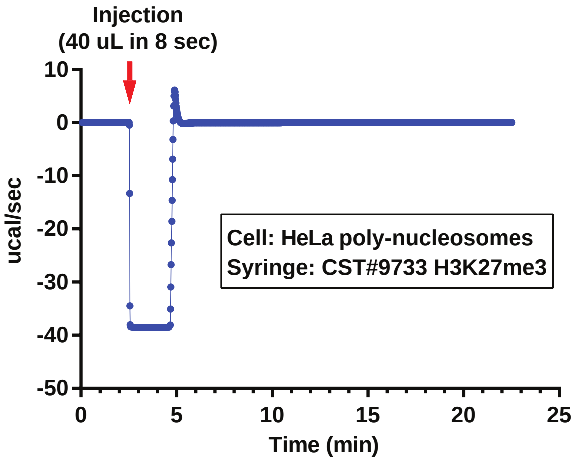

SI-Fig. 8: **Equilibration time.** To evaluate whether the antibody-chromatin complex formation behaves as an equilibrium binding reaction, we performed a single injection isothermal titration calorimetry (ITC) experiment under the same conditions as our ChIP reaction. H3K27me3 antibody and HeLa chromatin were each diluted in ChIP buffer 1 and completely combined with a single injection on the microcalorimeter. After approximately three minutes, the binding reaction reached equilibrium.

siQ-ChIP:

siQ-ChIP input files were prepared as described above and the "outfile.bed" data was processed using the tools at https://github.com/BradleyDickson/siQ-ChIP

Chromatin-Antibody binding Assay:

Antibody+chromatin interaction was measured (Figure 8) on a MicroCal PEAQ-ITC (Malvern) at 4°C to investigate relevance of equilibrium models. HeLa polynucleosomes (EpiCypher, 16-0003) or H3K27me3 antibody (CST #9733) were diluted in ChIP Buffer 1 to 0.75 ug in 280 uL (cell volume) or 5 uL into 40 uL (syringe), respectively. After an initial delay of 150 seconds, a single 40 uL injection was performed over 8 seconds, followed by an equilibration period. Equilibrium is reached in approximately three minutes, showing that IP conditions are compatible with equilibrium binding models.

Existence and determination of IP isotherms:

For each point on the isotherm (binding curve), 2 µg of purified HeLa polynucleosomes (EpiCypher, 16-0003) were digested for 5 min at 37^{circ}C with (without for poly) 1.5 units of MNase (Worthington) after addition of CutSmart buffer (New England Biolabs, 1X final) and CaCl2 (40 mM final). Digestion was quenched with EDTA (50 mM final), brought to a volume of 200 µL in binding buffer (25 mM HEPES pH 7.5, 100 mM NaCl, 0.05% NP-40, 1% BSA), and added to 25 µL Protein A magnetic beads (Invitrogen ,10001D) pre-complexed with varying volume of antibodies (H3K36me2, EMD Millipore 07-369-I; H3K27ac, Active Motif 39133; H3K27me3, Cell Signaling Technology 9733). Chromatin was next immunoprecipitated for 10 minutes at room temperature, washed twice with 500 µL of binding buffer, and chromatin was eluted in binding buffer + 1% SDS. Protein was digested by 10 μg of proteinase K at 50^{circ}C for 30 minutes, followed by DNA clean up on Qiagen MinElute columns, eluted in 30 μL of TE buffer, and quantified by Qubit fluorimeter (dsDNA HS).

Peptide microarray of H3K27me3 antibody (CST #9733) and H3K9me3 antibody (Active Motif 39161, Lot# 14418003)

Arrays were fabricated and antibodies were arrayed as described.[11] Briefly, each antibody was diluted 1:5000 and arrayed in a volume of 600 μL per subarray for 30 minutes at room temperature. Following washing, secondary antibody (anti-rabbit AlexaFluor 647, Invitrogen A21245) was diluted 1:10000 and the whole slide (subarray for each antibody) was incubated in 3 mL for 30 minutes at room temperature. Slide was washed and imaged at 20 um resolution (Innopsys InnoScan 1100AL). All array data were processed using ArrayNinja[12].

TABLE I: Required parameters for siQ-ChIP. Superscript a indicates measurements made by sequencing core or service, rather than by the ChIP practitioner. In ρ, 660 g/mol is the average base pair molar mass. This is converted to nanogram scale in application.

Symbol	Definition	Measurement	Veh1	Veh2	EPZ1	EPZ2	Sample Equation (e.g. Veh1)
$\mathcal{F}$	Fraction of IP taken to library	Total IP mass (ng)	55.5	56.6	24.2	35.2	= 10.0/55.5
		IP mass into library (ng)	10.0	10.0	10.0	10.0	
$\mathcal{F}_{in}$	Fraction of input taken to library	Total input mass (ng)	57.5	57.5	50.5	50.5	= 10.0/57.5
		Input mass into library (ng)	10.0	10.0	10.0	10.0	
$\mathcal{F}^L$	Fraction of IP library sequenced	^{a}IP library conc. (nM)	55.75	56.60	42.80	59.26	= (10.0*2.0)/(55.75*20.0)
		Total IP library volume (μL)	20.0	20.0	20.0	20.0	
		^{a}Normalized input library conc (nM)	10.0	10.0	10.0	10.0	
		^{a}Volume of normalized IP library loaded into sequencer (μL)	2.0	2.0	2.0	2.0	
$\mathcal{F}^L_{in}$	Fraction of input library sequenced	^{a}Input library conc. (nM)	121.76	121.76	85.15	85.15	= (10.0*4.0)/(121.76*20.0)
		Total input library volume (μL)	20.0	20.0	20.0	20.0	
		^{a}Normalized input library conc. (nM)	10.0	10.0	10.0	10.0	
		^{a}Volume of normalized input library loaded into sequencer (μL)	4.0	4.0	4.0	4.0	
$\frac{v_i}{V-v_i}$	Fraction of chromatin taken for input	Volume taken for input (μL)	100.0	100.0	100.0	100.0	= 100.0/(1000.0-100.0)
		Total volume of chromatin (μL)	1000.0	1000.0	1000.0	1000.0	
ρ	Ratio of captured IP libraryconc. to the expected library conc.	IP mass into library (ng)	10.0	10.0	10.0	10.0	$\rho = \frac{55.75}{\left(\frac{10^{-9}*10*2^{11}}{(691*660)(20*10^{-6})}\right)}$
		PCR amplifications (C_t)	11.0	11.0	11.0	11.0	
		Total IP library volume (μL)	20.0	20.0	20.0	20.0	
		^{a}IP library conc. (nM)	55.75	56.60	42.80	59.26	
		^{a}Average IP fragment length (bp)	691.0	735.0	648.0	676.0	
ρ_{in}	Ratio of captured input libraryconc. to the expected library conc.	Input mass into library (ng)	10.0	10.0	10.0	10.0	$\rho_{in} = \frac{121.76}{\left(\frac{10^{-9}*10*2^{11}}{(367*660)(20*10^{-6})}\right)}$
		PCR amplifications (C_t)	11.0	11.0	11.0	11.0	
		Total input library volume (μL)	20.0	20.0	20.0	20.0	
		^{a}Input library concentration (nM)	121.76	121.76	85.15	85.15	
		^{a}Average input fragment length (bp)	367.0	367.0	380.0	380.0	

[a] These measurements are made by the person running the sequencer and are not routinely collected by the ChIP practitioner

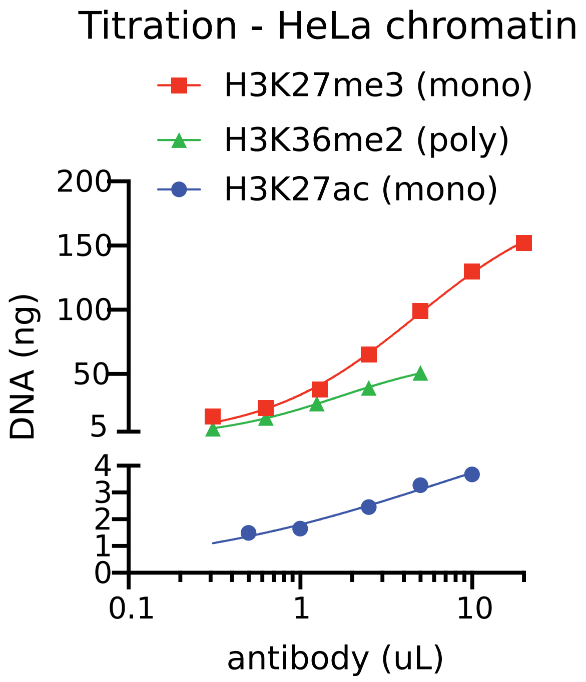

SI-Fig. 9: **IP isotherms:** The binding isotherm for antibody and chromatin exists for any interacting antibody-chromatin pair and gives rise to the quantitative scale of siQ-ChIP. Figure 8 shows equilibration of a single point on an isotherm.

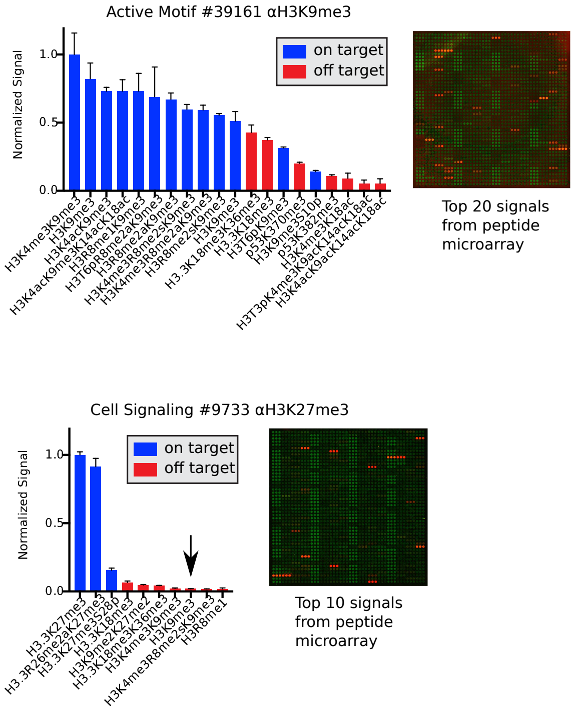

SI-Fig. 10: **Microarray profiles:** The microarray suggests low affinity cross-reaction for the respective antibody targets.

* **bradley.dickson@vai.org**, scott.rothbart@vai.org

[1] Mathai Mammen, Seok-Ki Choi, and George M Whitesides. Polyvalent interactions in biological systems: implications for design and use of multivalent ligands and inhibitors. *Angewandte Chemie International Edition*, 37(20):2754–2794, 1998.

[2] Adrian T Grzybowski, Zhonglei Chen, and Alexander J Ruthenburg. Calibrating chip-seq with nucleosomal internal standards to measure histone modification density genome wide. *Molecular cell*, 58(5):886–899, 2015.

[3] Rohan N Shah, Adrian T Grzybowski, Evan M Cornett, Andrea L Johnstone, Bradley M Dickson, Brandon A Boone, Marcus A Cheek, Martis W Cowles, Danielle Maryanski, Matthew J Meiners, et al. Examining the roles of h3k4 methylation states with systematically characterized antibodies. *Molecular cell*, 72(1):162–177, 2018.

[4] Shingo Nishikori, Takamitsu Hattori, Stephen M Fuchs, Norihisa Yasui, John Wojcik, Akiko Koide, Brian D Strahl, and Shohei Koide. Broad ranges of affinity and specificity of anti-histone antibodies revealed by a quantitative peptide immunoprecipitation assay. *Journal of molecular biology*, 424(5):391–399, 2012.

[5] Yong Zhang, Tao Liu, Clifford A Meyer, Jérôme Eeckhoute, David S Johnson, Bradley E Bernstein, Chad Nusbaum, Richard M Myers, Myles Brown, Wei Li, et al. Model-based analysis of chip-seq (macs). *Genome biology*, 9(9):R137, 2008.

[6] Kaifu Chen, Zheng Hu, Zheng Xia, Dongyu Zhao, Wei Li, and Jessica K Tyler. The overlooked fact: fundamental need for spike-in control for virtually all genome-wide analyses. *Molecular and cellular biology*, 36(5):662–667, 2016.

[7] Scott B Rothbart, Bradley M Dickson, Jesse R Raab, Adrian T Grzybowski, Krzysztof Krajewski, Angela H Guo, Erin K Shanle, Steven Z Josefowicz, Stephen M Fuchs, C David Allis, et al. An interactive database for the assessment of histone antibody specificity. *Molecular cell*, 59(3):502–511, 2015.

[8] Ben Langmead and Steven L Salzberg. Fast gapped-read alignment with bowtie 2. *Nature methods*, 9(4):357, 2012.

[9] Thomas S Carroll, Ziwei Liang, Rafik Salama, Rory Stark, and Ines de Santiago. Impact of artifact removal on chip quality metrics in chip-seq and chip-exo data. *Frontiers in genetics*, 5:75, 2014.

[10] Aaron R Quinlan and Ira M Hall. Bedtools: a flexible suite of utilities for comparing genomic features. *Bioinformatics*, 26(6):841–842, 2010.

[11] Evan M Cornett, Bradley M Dickson, and Scott B Rothbart. Analysis of histone antibody specificity with peptide microarrays. *JoVE (Journal of Visualized Experiments)*, (126):e55912, 2017.

[12] BM Dickson, EM Cornett, Z Ramjan, and SB Rothbart. Arrayninja: an open source platform for unified planning and analysis of microarray experiments. In *Methods in enzymology*, volume 574, pages 53–77. Elsevier, 2016.
